# Identification of ZFTA as a Novel KLHL20 Substrate and Mechanistic Insights Into Fuzzy Binding of Disordered Peptides via Biosensor Analysis and Computational Modelling

**DOI:** 10.1002/cbic.70237

**Published:** 2026-02-28

**Authors:** Nadine E. M. Myers, Joanna Whittaker, Marie Elodie Hélène Cadot, Julia K. Varga, Marcel Diallo, Jakob Nilsson, Anders Bach, Anja Sandström, Ora Schueler‐Furman, U. Helena Danielson

**Affiliations:** ^1^ Dept. of Chemistry for the Life Sciences Uppsala University Uppsala Sweden; ^2^ Beactica Therapeutics AB Uppsala Sweden; ^3^ Dept. of Drug Design and Pharmacology Faculty of Health and Medical Sciences University of Copenhagen Copenhagen Denmark; ^4^ Dept. of Microbiology and Molecular Genetics Institute for Biomedical Research Israel‐Canada Faculty of Medicine The Hebrew University of Jerusalem Jerusalem Israel; ^5^ Novo Nordisk Foundation Center for Protein Research ICMM, University of Copenhagen Copenhagen Denmark; ^6^ Center for Epigenetic Cell Memory Danish Cancer Institute Copenhagen Denmark; ^7^ Dept. of Medicinal Chemistry Uppsala University Uppsala Sweden; ^8^ Science for Life Laboratory Uppsala University Uppsala Sweden

**Keywords:** biosensor, computational design, Kelch, KLHL20, stapled peptide

## Abstract

Interactions between peptides based on a region in the zinc finger translocation associated (ZFTA) protein and the Kelch domain of Kelch‐like protein 20 (KLHL20^Kelch^) have been characterised by biosensor analysis, supported by AlphaFold2‐based structure predictions of peptides bound to the protein. Residues critical for the interaction were identified. The analysis showed that all peptides exhibited relatively weak and complex interactions with KLHL20^Kelch^. The original ZFTA peptide had a much higher affinity for KLHL20^Kelch^ than for the Kelch domain of KLHL12 (KLHL12^Kelch^), indicating a specificity for KLHL20^Kelch^. The estimated *K*_D_^app^ of 35 µM was like that for a 21‐mer peptide derived from death‐associated protein kinase 1, a known KLHL20 substrate. Removal of flexible C‐terminal residues generated a 12‐mer, predicted to form a stable helix. This reduced the affinity 100‐fold. Removal of N‐terminal residues resulted in a 10‐mer predicted to be flexible, which had a similar affinity as the original 16‐mer. The similar affinities for peptides representing different regions of ZFTA suggest that the recognition is feature specific rather than sequence specific. The interaction mechanism reflects “fuzzy binding”, consistent with the role of KLHL20 as an adaptor protein in the ubiquitination of disordered protein substrates by Cullin‐3 E3 ubiquitin ligase.

## Graphical abstract

An AlphaFold 2 model of a peptide derived from zinc finger translocation associated (ZFTA) protein in complex with the Kelch domain of KLHL20 (KLHL20^Kelch^). Sensorgrams from surface plasmon resonance biosensor experiments of the interaction between a ZFTA‐derived peptide with immobilised KLHL20^Kelch^ shows that the interaction is complex and has a millimolar affinity. A fuzzy binding mechanism describes the complexities and specificity of the interaction.

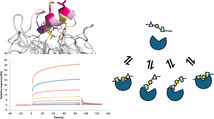

## 1 Introduction

Kelch like protein 20 (KLHL20) is part of the ubiquitin proteasome system (UPS) which controls cellular protein levels via protein degradation. This process occurs in three stages, catalysed by E1, E2, and E3 ubiquitin ligases, respectively, and multiple accessory proteins. E3 ligases capture proteins destined for degradation and positions them within the complex for ubiquitination. KLHL20 forms a multisubunit complex with Cullin‐3 E3 ubiquitin ligase (Cul3), RING‐Box 1 (RBX1), and the ubiquitin‐like protein neural precursor cell expressed developmentally down‐regulated protein 8 (NEDD8) [1, 2]. KLHL20 serves as an adaptor protein within the Cullin‐RING ubiquitin ligase complex. The Kelch domain is responsible for substrate recognition. However, the specificity of the recognition is not understood as KLHL20 is involved in the ubiquitination of a range of proteins involved in several cellular processes, but with no known common consensus sequence [2, 3, 4, 5, 6, 7]. Degradation of the tumour suppressor proteins, death‐associated protein kinase 1 (DAPK1) and promyelocytic leukaemia protein (PML) have been of particular interest and KLHL20‐mediated PML degradation appears to have a key role in prostate tumour progression [2, 3, 6, 7, 8, 9].

Our aim was initially to identify KLHL20 ligands with the potential to be developed into compounds that can regulate the concentration of KLHL20 substrates by interfering with their ubiquitination and subsequent degradation via the UPS. Ligands blocking KLHL20 substrate interactions are expected to reduce KLHL20 substrate ubiquitination, whilst ligands incorporated in bifunctional compounds targeting both KLHL20 and a KLHL20‐substrate, are expected to promote ubiquitination of the substrate by induced proximity. However, as the project evolved, the aim shifted to establishing the substrate recognition mechanism and specificity of KLHL20.

Previous studies with KLHL20^Kelch^ have identified a “LPDLV” motif in DAPK1‐derived peptides to be critical for their interaction, showing that substrate derived peptides may serve as starting points for inhibitor design [9]. This motif has later been exploited in the design of a macrocyclic peptide, combined with a bromodomain and extra‐terminal domain (BET) inhibitor, and further evolved into compounds capable of degrading BET family BRD proteins in multiple cancer cell lines, showing a robust proof‐of‐principle for the induced proximity approach [10].

Here, we turned to a 16‐mer peptide encompassing amino acids 426–441 of zinc finger translocation associated protein (ZFTA (also known as C11or f95)) identified via proteomic peptide‐phage display (ProP‐PD) (Ylva Ivarsson, Uppsala University, personal communication) to bind the Kelch domain of KLHL20 (KLHL20^Kelch^). Notably, this peptide had little resemblance to the DAPK1 peptide sequence. In addition, we have not attempted to establish the biological relevance of ZFTA as a KLKL20 substrate, but here simply used the 16‐mer peptide as a tool suited for our current studies.

We established a surface plasmon resonance (SPR) biosensor assay for KLHL20 lead discovery using the Kelch domain to confirm and characterise the interaction between the ZFTA peptide and KLHL20^Kelch^. Initial experiments with the original ZFTA^426–441^ peptide showed it had a weak affinity for KLHL20^Kelch^. To better understand critical residues and the mechanism for the interaction, a series of analogues were designed based on computational structure predictions and modelling. The interactions of the synthesised peptides with KLHL20^Kelch^ were subsequently characterised using the SPR biosensor assay. All peptides exhibited complex interactions with KLHL20^Kelch^, which required exploration of different experimental procedures and approaches for data analysis. The analysis revealed that most analogues had lower affinities than the original peptide, which led us to revisit the initial structural models and the design of a new peptide. It revealed that both N‐ and C‐terminal regions in the peptide can independently be part of substrate recognition.

This study suggests that KLHL20 substrate binding involves recognition of structural features that may be localised in several regions in the substrate. This is similar to the binding of the transcription factor NF‐E2‐related factor 2 (Nrf2) to the Kelch domain in Kelch‐like ECH‐associated protein 1 (KEAP1) [11]. However, the exact mechanism of action may be a variation on the broad theme of the different modes of multivalent substrate‐E3 interactions that have been described and reviewed [12, 13]. The dynamic features of the substrate binding site in the KLHL family have practical consequences for the discovery of drug candidates.

## 2 Results

### 2.1 Interaction Analysis of ZFTA and DAPK1 Peptides

**FIGURE 1:**
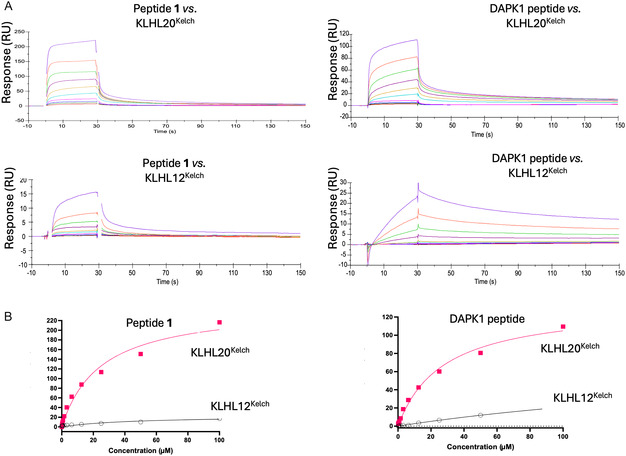
SPR biosensor data for 16‐mer ZFTA peptide (**1**) and 21‐mer DAPK1 peptide interacting with KLHL20^Kelch^ and KLHL12^Kelch^ sensor surfaces. Note the different *y* axis scales. (A) Sensorgrams for peptides injected as analytes in 1:1 dilution series starting from 100 µM. (See Figure S1 for global fitting using 1:1 and 2‐state models.) (B) Pseudo‐steady state analysis of data in A, based on signals for report points at the end of injections, plotted against concentrations of peptide **1** (left) and DAPK1 (right) for KLHL20^Kelch^ (red squares) and KLHL12^Kelch^ (black circles).

The interaction between the ProP‐PD derived 16‐mer ZFTA peptide (YLMELDGGRRGLVCGV, peptide **1**) and KLHL20^Kelch^ was characterised using an SPR biosensor assay where concentration series of peptides were injected as analyte over immobilised KLHL20^Kelch^ or KLHL12^Kelch^ (Figure 1). A 21‐mer DAPK1 peptide that included the critical LPDLV motif (LLAMNLGLPDLVAKYNTSNGA) was used as a reference.

The experiment showed a concentration‐dependent response for peptide **1** (Figure 1A, top left). Global nonlinear regression analysis of sensorgrams using a 1:1 interaction model did not fit the data well but allowed an *apparent* *K*_D_ (*K*_D_^app^) = 8 µM to be estimated (Figure S1). A marginally better fit was obtained with a 2‐state model. No other tested models were superior. Analysis of steady state report points taken at the end of injection also showed a poor fit with a 1:1 interaction model, with no obvious saturation being reached. However, the analysis estimated the *K*_D_^app^ to be ca. 35 µM (Figure 1B, left), corroborating that the affinity of the interaction was in the micromolar range. However, the exact interaction mechanism could not be established from these fits.

The interaction between the 21‐mer DAPK1 peptide and KLHL20^Kelch^ was similar (Figure 1A, top right and Figure S1) with *K*_D_^app^ estimates of 12 µM from a global analysis of the sensorgrams using a 1:1 model, and 34 µM from the steady state analysis of report points (Figure 1B, right). Although the analyses suggest that DAPK1 and peptide **1** have similar *K*_D_ values, peptide **1** is concluded to have a slightly higher affinity. This is based on the sensorgrams in Figure 1, which show that the signals for peptide **1** are higher than those for DAPK1, when injected at the same concentration. The differences cannot be attributed to differences in molecular weights of the peptides since peptide **1** has the highest molecular weight (1779 Da versus 2175 Da for DAPK1).

The specificity of the peptides for KLHL20^Kelch^ were analysed using the Kelch domain of another member of the Kelch like protein family, KLHL12 (KLHL12^Kelch^). Both peptides showed weak interactions toward KLHL12^Kelch^, with the weakest binding seen for peptide **1** indicating that it is specific for KLHL20^Kelch^ rather than just a Kelch domain (Figure 1).

**FIGURE 2:**
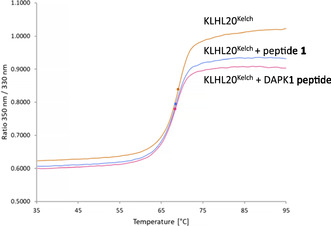
Thermal shift analysis of KLHL20^Kelch^ (6 µM) in the presence of 50 µM ZFTA peptide (**1**) or 100 µM DAPK1 peptide, monitored using intrinsic fluorescence.

The potential stabilising effects of the ZFTA (**1**) and DAPK1 peptides on the structural integrity of KLHL20^Kelch^ was assessed using a solution based thermal shift assay (TSA) and the highest concentrations of the peptides that could be used. It showed that KLHL20^Kelch^ appears to be folded, with a melting temperature (*T*_m_) of 69°C that was not significantly affected by binding of either peptide (Figure 2).

### 2.2 Modelling and Rational Design of ZFTA Analogues

**FIGURE 3:**
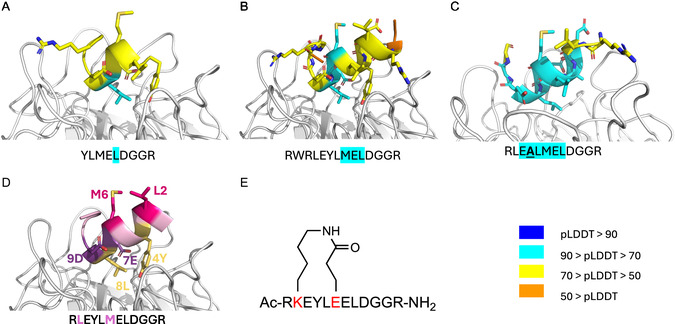
Structures of AF2 predictions of ZFTA peptide variants in complex with KLHL20^Kelch^. (A) N‐terminal region of peptide **1**, (B) peptide **2**, and (C) peptide **4**. In panels (A–C), side chains in the structure are colour coded according to their pLDDT while residues in the sequences underneath are highlighted in turquoise if their pLDDT was between 70 and 90. (D) The peptide scaffold used to generate the stapled peptide (peptide **8**). The two substituted side chains that were stapled via a peptide bond are in magenta. Hydrophobic residues in the core of the interaction are in yellow and charged residues in purple. (E) Chemical structure of stapled peptide (peptide **8**) with stapled residues in red.

A structure‐based computational approach was taken to identify critical residues in the ZFTA peptide for its interaction with KLHL20^Kelch^ and design analogues with potentially higher affinities. As a starting point, the ZFTA peptide–KLHL20^Kelch^ complex was modelled using AlphaFold2‐multimer‐v3 (AF2) [14, 15] (Figure 3).

However, docking of the original 16‐mer peptide resulted in a heavily clashing unconfident model (pLDDT < 50 along the peptide). It was hypothesised that cropping the peptide into two shorter peptides (N‐terminal fragment: YLMELDGGRR and C‐terminal fragment: GGRRGLVCGV, peptide **9**) could increase the confidence of the model. Indeed, modelling the N‐terminal fragment of the peptide resulted in a more confident conformation (pLDDT 50–70), with a few side chains pointing into the binding pocket (Figure 3A). The C‐terminal part of the peptide was modelled in an extended conformation with pLDDT 50–70 (Figure S2). Given that the N‐terminal peptide contains a helix turn in the model, we considered if additional N‐terminal amino acids could potentially help stabilise the helix. Based on the ZFTA sequence, the peptide was extended N‐terminally by 5 residues (Figure 3B). This appeared to stabilise the helix in the models. Initially, we did not focus on the C‐terminal fragment but returned to it later (see below).

To identify critical side chains in the N‐terminus of the peptide for KLHL20^Kelch^ interaction, the effect of alanine substitutions was modelled (Table 1) [16]. Out of nine amino acid substitutions, one was predicted to strengthen the interaction whilst three were predicted to disrupt it. Five residues did not have side chains pointing into the binding site and were consequently considered unlikely to be important for the interaction. These predictions were used to prioritise the synthesis of peptides **4**–**7** (see Table 2).

**TABLE 1:** Predicted effect of alanine substitutions in the N‐terminal fragment of peptide 1 (YLMELDGGR), used as template for alanine scanning. Peptides synthesised with the corresponding substitution are specified.

Residue #	Substitution	Predicted effect	Peptide
1	Y to A	Disrupt	**4**
2	L to A	None^a^	
3	M to A	None	
4	E to A	Strengthen	**5**
5	L to A	Disrupt	**6**
6	D to A	Disrupt	**7**
7	G to A	None	
8	G to A	None	
9	R to A	None	

a None means that the residue is not in the binding interface.

**TABLE 2:** Designed and analysed ZFTA derived peptide analogues.

Peptide #	Sequence	Structural feature	MW, Da	Binding efficiency, RU/µM	*K*_D_^app^, mM
ZFTA	‐ *RLEYLMELD* *GGR*RGLVCGV ‐	Protein seq.	—	—	—
**1**	*YLMELD* *GGR*RGLVCGV	Orig. peptide	1779	2.8	0.030
**2**	*RLEYLMELD* *GGR*	Ext. N‐term.	1493	0.33	0.29
**3**	*RLEYLMELD*	Rem. C‐term.	1222	nd	nd
**4**	*RLE* ***A*** *LMELD* *GGR*	Y4A	1403	—	0.16
**5**	*RLEYLM* ***A*** *LD* *GGR*	E7A	1434	—	0.80
**6**	*RLEYLME* ***A*** *D* *GGR*	L8A	1451	—	50
**7**	*RLEYLMEL**A**GGR*	D9A	1449	—	≫100
**8**	*R**K**EYL**E**ELDGGR*	Stapled K to E	1488	2	0.17
**9**	*GGRRGLVCGV*	Rem. N‐term.	1014	0.52	0.26

All peptides had an acetylated N‐terminus and an amidated C‐terminus. The N‐terminal region taken from peptide **2** is highlighted in italics, the C‐terminal region defined by peptide **9** is underlined. Introduced alanine residues are in bold (**A**), substituted and stapled amino acids are in bold (**K** and **E**) (see Figure 3E). Binding efficiency was calculated from the initial slope of the steady‐state graphs [17]. K_D_^app^ values are from regression analysis of steady state data using a 1:1 interaction model. See Figure S1 and S3 for global fits of sensorgrams for peptides **1** and **2**, and DAPK1. Nd: No detected interaction. ‐: Not estimated.

Peptide **1** was used as a starting point for designing an optimised ligand for KLHL20^Kelch^. The designed and tested peptides are presented in Table 2. Peptide **2** was designed by adjusting the N‐ and C‐termini according to the modelling outlined above. Specifically, three residues (RLE‐) from the original sequence were added at the N‐terminus based on the hypothesis they would stabilise the helix. The C‐terminus was truncated in order to remove flexible side chains predicted to not be part of the helical structure or interacting with the domain. It was noted that an arginine at the C‐terminal end of the helix is positioned within a pocket on the protein, so this residue was selected to potentially anchor the C‐terminus of the peptide in the pocket. Peptide **3** is a C‐terminally truncated variant with ‐GGR removed. It was designed to test the hypothesis that this peptide would be unable to form a helix, and its affinity would therefore be lower than that of peptide **2**.

Peptides **4**–**7** have key residues replaced by alanine, while peptide **8** is a stapled peptide. Its design was based on the observation that residues L2 and M6 were predicted to point away from the protein (Figures 3B–D). L2K and M6E substitutions were made, and the side chains were connected via an amide bond, generating a lactam‐stapled peptide (Figure 3E). The aim was to potentially increase the structural stability of the peptide and thereby its affinity for KLHL20^Kelch^.

Designed peptides were synthesised and purified to > 95% (See Figure S4 for identity and purity controls).

### 2.3 Interaction Analysis of N‐Terminal ZFTA Peptide Analogues

The interactions between the designed N‐terminally extended ZFTA analogues and immobilised KLHL20^Kelch^ were assessed using the SPR biosensor assay. The data for these peptides was influenced by small variations in experimental design and procedures, affecting sensor surface functionality. Storage and handling of the peptides was also a critical factor. Analytical comparisons were therefore only done between data from same the experiment and the same sensor surface configuration. In addition, affinities were generally low, as can be deduced from the steady state vs. concentration plots, which are either linear or have minimal curvature with no sign of saturation at the highest concentrations used (typically 200 µM). Limited solubility of the peptides prevented experiments at higher concentrations.

**FIGURE 4:**
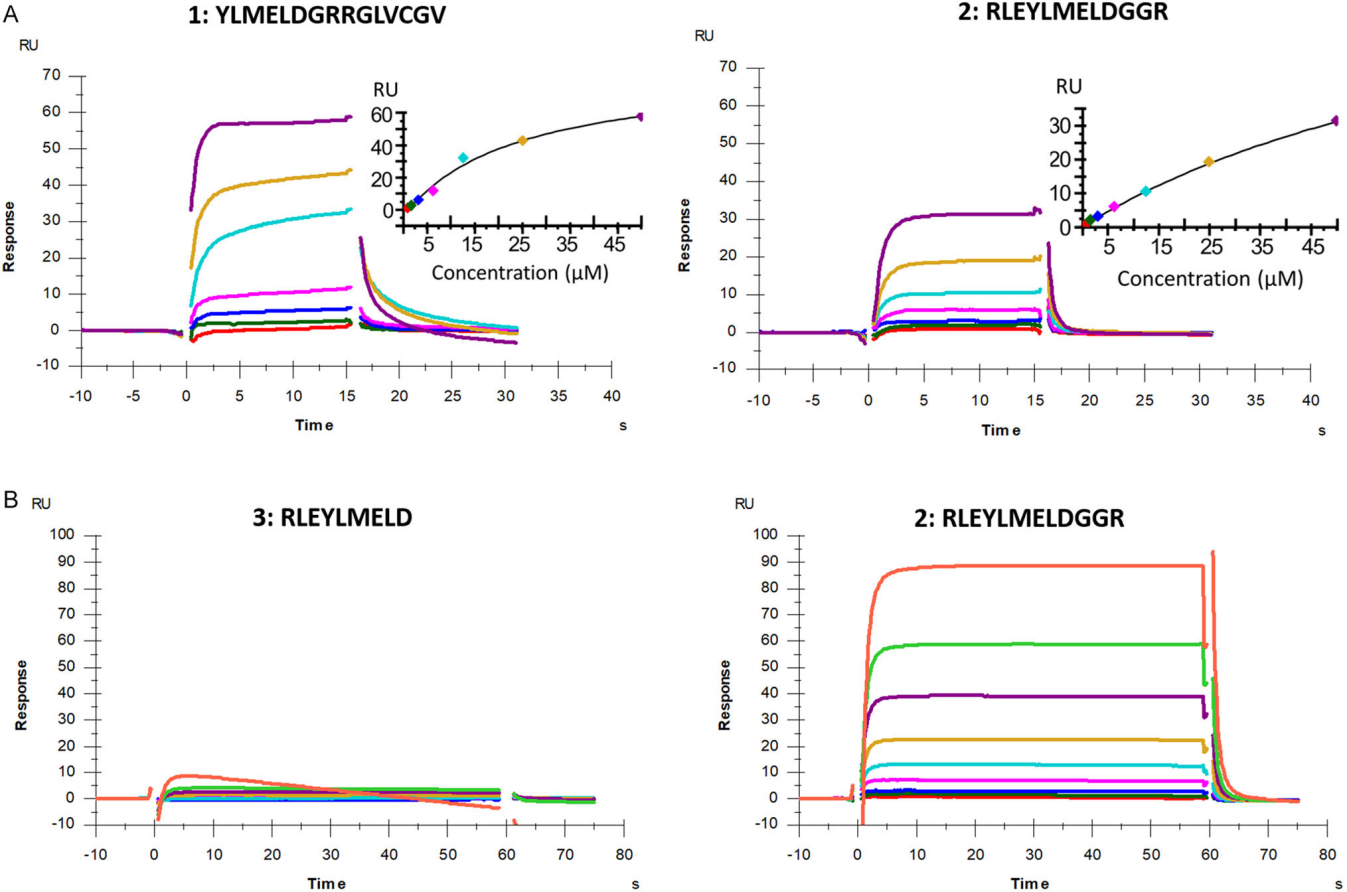
SPR biosensor data for peptides **1**, **2**, and **3** interacting with KLHL20^Kelch^. Top panels (A) are from an experiment with 15 s injection times and top concentrations of 50 µM, bottom panels (B) are from an experiment with 60 s injection times and top concentrations of 200 µM. Insets show signals for report points at the end of injections, plotted against peptide concentration.

Sensorgram comparisons show that peptide **2** has a lower affinity than peptide **1** (Figure 4, top panels and Figure S3) and no interaction was seen for peptide **3**, as predicted (Figure 4, bottom panels). Note that differences in signal magnitude is partly a consequence of differences in peptide molecular weights (Table 2). Global nonlinear regression analysis of the interactions for these peptides was done using two different interaction models, as shown in Figure S3. The standard 1:1 model fitted poorly, revealing the interactions to be more complex. However, there was no significant improvement when instead using a 2‐state model. The 1:1 model was therefore used to roughly estimate affinities. For peptide **1**, the estimated *K*_D_^app^‐value was 24 µM while that for peptide **2** was 10 mM. The differences in affinity may be attributed to a faster rate of association and slower rate of dissociation, although these could not be quantified due to the complex interaction. Due to the limitations of regression analysis for complex interactions, the binding efficiencies [17] of the peptides were also estimated from the initial slope of the steady‐state graphs and *K*_D_^app^ values were estimated from steady state data using a 1:1 interaction model. (Table 2). Taken together, all estimates of the affinities of the peptides indicate that peptide **1** has a higher affinity than peptide **2**.

**FIGURE 5:**
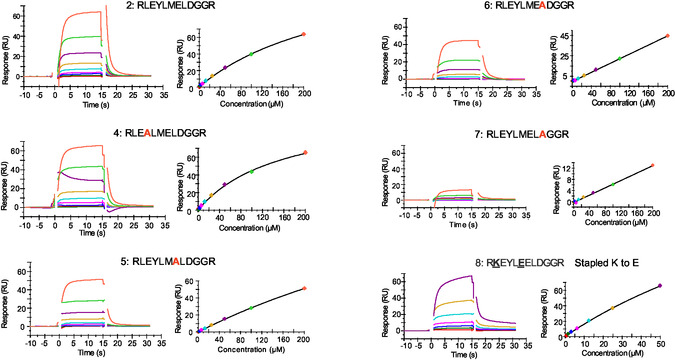
SPR biosensor data for designed peptides (Table 2) interacting with KLHL20^Kelch^. Sensorgrams and steady‐state graphs (based on report points taken at the end of injections) for peptides injected in concentration series (1:1 – dilutions from a top concentration of 200 µM for all peptides except stapled peptide **8**, which had a top concentration of 50 µM). Gaps in the data are a consequence of the removal of signal spikes which are due to pressure changes in the system before and after injections. These sensorgrams with fits from nonlinear regression analysis are also shown in Figure S5. Data for the corresponding experiment with 60 s injections are shown in Figure S6. Data for peptide (**8**) injected at 15 and 60 s injections in Figure S8.

To identify critical residues of the interactions, and explore the effect of stapling peptide **2**, the interactions between peptide **2**, the corresponding alanine substituted peptides (peptides **4**–**7**) and stapled peptide **8** with KLHL20^Kelch^ were analysed using the biosensor assay (Figure 5). All data sets in this figure are from the same experiment, using the same sensor surface to assure that observed differences are not due to slight variations between surfaces and peptide samples in different experiments. The same data with overlaid theoretical curves from non‐linear regression data analysis using a 2‐state model is shown in Figure S5, highlighting the complexity of all interactions. Similar fits were obtained using a 1:1 model (not shown). Peptides were initially tested at a maximal concentration of 50 µM and with injection times of 15 s. Later, the maximal concentration was increased to 200 µM and the injection times increased to 60 s, allowing interactions to reach a steady state to potentially enable estimation of *K*_D_‐values (Figure S6). However, none of the data sets were well fitted using any of the standard models for interactions. The effect of alanine substitutions was therefore based on comparisons of sensorgrams, steady state graphs, and BE estimates.

The experimental data for the alanine substituted peptides partially agreed with the computational predictions (Tables 1 and 2), supporting the hypothesised binding conformation of the peptide. The experimental data for the Y4A substitution (peptide **4**), initially did not match the prediction, as it was predicted to disrupt the interaction but appeared to strengthen it. Indeed, a model of the alanine‐substituted peptide resulted in increased confidence of the predicted position of this substituted residue (Figure 3C). Similarly, the substitution represented by peptide **5** was expected to strengthen the interaction, but it was found to become weaker. Peptides **6** and **7** were both predicted to include changes that would disrupt the interaction. This was observed, with a very clear effect for peptide **7** in which an aspartic acid was substituted by alanine. This destroyed the interaction almost entirely, exposing this residue as critical for the interaction. This correlates with the effect of removing residues at the C‐terminus of the original peptide, yielding peptide **3**. In contrast, the stapled peptide (**8**) was found to have a similar affinity as peptide **2**, supporting the hypothesis that the residues incorporated in the staple did not interact directly with the protein.

Taken together, all peptides had weak and complex interactions with KLHL20^Kelch^. Addition of three residues at the N‐terminus and truncating the C‐terminus by seven amino acids, resulted in a lower affinity than the original 16‐mer ZFTA_426–441_ peptide. Further modifications resulted in minor or major additional reductions in the affinity.

### 2.4 Interaction Analysis of C‐Terminal ZFTA Peptide Analogue

**FIGURE 6:**
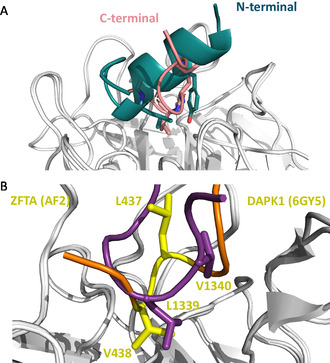
(A) C‐ and N‐terminal regions of peptide **1** (pink and teal, resp.) are predicted to bind to the same pocket in KLHL20^Kelch^, mutually excluding one another. (B) Superimposition of predicted C‐terminal regions of ZFTA peptide **1** (yellow and orange) and crystal structure of DAPK1 (purple, 6GY5.PDB) in complex with KLHL20^Kelch^ showing V438 in ZFTA bound in the same pocket as L1339 DAPK1 (numbering from complete protein sequences).

Based on the low affinities of the peptides derived from the N‐terminal region of the original ZFTA peptide, we postulated that the C‐terminal part of the ZFTA_426–441_ peptide was important for the interaction, despite its predicted disorder and the difficulties in modelling this region of the peptide. It was envisioned that the C‐terminal region could transiently interact with the substrate binding pocket—strengthening the interaction—although most of the affinity would be due to interaction with the helical N‐terminus. We therefore designed a peptide corresponding to the isolated C‐terminal region (GGRRGLVCGV, peptide **9**). Albeit of lower confidence, the structural model suggested that peptide **9** may act as a second recognition region, binding at the same pocket as the N‐terminal region (Figure 6A).

A comparison between peptide **1** in this model and the crystal structure of the DAPK1 peptide, predicted that the first valine in the ZFTA “GLVCGV” C‐terminal region overlaps with the second leucine in the critical “LPDLV” DAPK1 motif (Figure 6B). The low confidence of modelling the C‐terminus of peptide **1** may reflect that it is flexible even upon binding, with multiple rapidly interchanging conformations.

**FIGURE 7:**
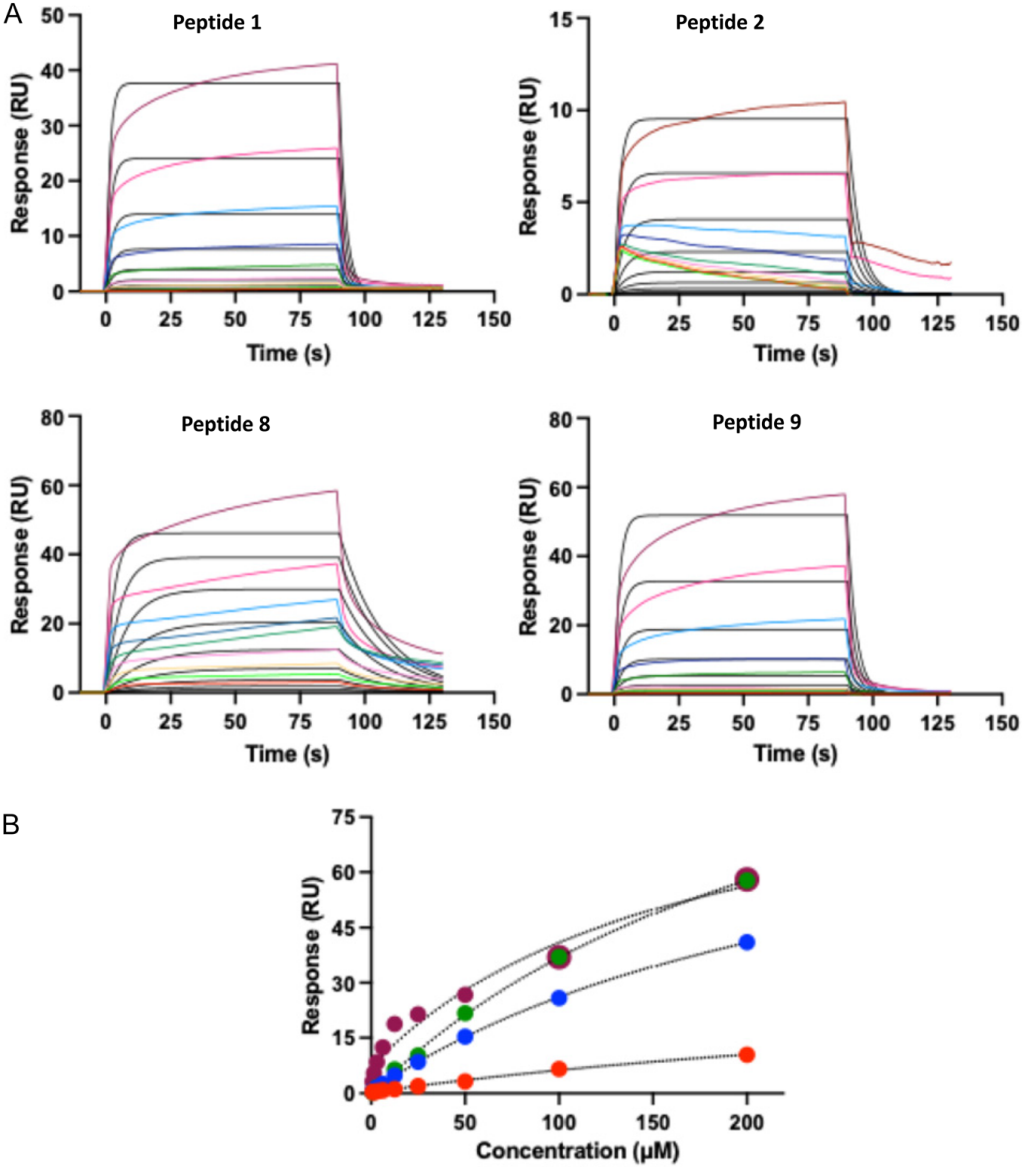
SPR biosensor data. (A) Sensorgrams for peptides **1**, **2, 8**, and **9**. injected as analytes in 1:1 dilution series from 200 µM with predicted curves using a 1:1 model (black). (B) Pseudo‐steady state analysis of data in A, based on signals for report points at the end of injections, vs. peptide concentrations. Peptide 1: blue spheres ; Peptide 2: red spheres : Peptide 8: purple spheres ; Peptide 9: green spheres . Dashed traces are from a fit using a 1:1 interaction model.

Peptide **9** was consequently acquired and tested for its ability to interact with KLHL20^Kelch^. The SPR biosensor data for this peptide, run together with peptides **1**, **2**, and **8** as references is shown in Figure 7. The new C‐terminal peptide (**9**) had a similar affinity as the stapled peptide, both with slightly higher affinities than peptide **1**.

### 2.5 Investigation of Interaction Mechanism

It was noted at the start of this project that the ZFTA and DAPK1 peptides had a complex interaction with KLHL20^Kelch^, since the sensorgrams were biphasic and did not reach steady state at high concentrations. This was confirmed in the global regression analysis since a 1:1 model was not suitable for the sensorgram series. An obvious option was a 2‐state model (Scheme 1 in Figure S7), which could be suitable if the binding of ligand could induce a conformational change in the protein. As shown for peptide **1**, the DAPK1 peptide and peptide **2** in Figures S1 and S3, there was no improvement with this model. Other more complex models were not any better (not shown).

**FIGURE 8:**
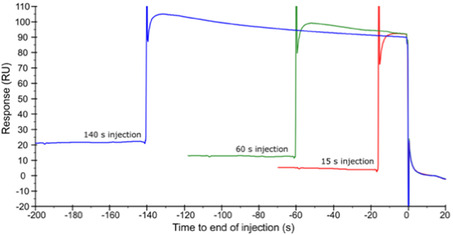
SPR biosensor data for peptide **8** injected at 50 µM and different injection times, aligned at the end of the injection (here set to time zero). See Figure S9 for data for all peptides.

To further explore the hypothesis that peptides may induce slow conformational changes in the protein upon binding, a set of experiments was carried out where peptides were injected for increasing intervals. Data for peptide **8** is shown in Figure 8, while a comparison of the concentration series of peptide **8** injected for 15 and 60 s is shown in Figure S8. An alternative representation of data from these time series injections, with the sensorgrams aligned at the start of the injection, is shown for peptide **8** and all other peptides in Figure S9.

None of these experiments show differences in the signal at steady state or the dissociation kinetics as a result of different injection times, indicating there is no effect of contact time. The slight drift and extensive noise seen after the dissociation of some peptides are likely due to small changes in the sensor surface rather than in the interaction itself since it is present both in the baseline and the steady state phase of the injection. Any observable differences are apparent in the binding levels, which directly correlate to the affinities rather than the differences in the interaction mechanism. Slow, rate‐limiting ligand‐induced conformational change of KLHL20^Kelch^ were consequently excluded as an explanation for their complex kinetic behaviour.

### 2.6 Structural Analysis

X‐ray crystallography experiments were initiated to determine the structure of KLHL20^Kelch^ in complex with peptides **1**, **2**, and **8**, while using the apo structure as a reference. Several protein constructs, strategies and conditions were used to produce protein and conduct initial crystallisation trials, including cocrystallisation with peptides via a published procedure for the KLHL20^Kelch^–DAPK1 complex [9]. Due to the lack of success, a systematic exploration of cryogenic freezing solutions was done, and TSA was used to evaluate the stability of the protein in each condition (Figure S10). The buffer condition resulting in the highest stability was chosen for a new series of crystallisation trials, with varying protein concentrations and incubation temperatures. Though the formation of transparent crystals was observed in many conditions, diffraction was only obtained with very low resolution (ca. 8 Å).

NMR was explored as an alternative method for structure determination. Although the yields of produced double isotopically labelled (^15^N and ^2^H) KLHL20^Kelch^ were very low (not shown), preliminary NMR experiments could be performed at a protein concentration of 200 µM. These confirmed that peptide **1** indeed binds to KLHL20^Kelch^ (Figure S11) but further experiments were not pursued due to the considerable resources required for production of larger amounts of labelled protein.

## 3 Discussion

This project was based on a peptide identified by phage display to be a potential ligand for KLHL20^Kelch^. As a first step, the specificity of the interaction between a 16‐mer ZFTA^426–441^ derived peptide (**1**) and KLHL20^Kelch^ was analysed. Since all KLHL proteins have a similar substrate binding pocket in the β‐propeller structure of the Kelch domain, a comparative analysis was done with KLHL12^Kelch^. The proteins are structurally similar but share little sequence homology. The stronger interaction between the ZFTA peptide and KLHL20^Kelch^ compared to KLHL12^Kelch^ indicates a specificity for KLHL20^Kelch^ and not simply for any Kelch domain containing protein.

The ZFTA derived peptide (**1**) had a higher affinity for KLHL20^Kelch^ than the DAPK1 peptide. The latter has previously been reported to have a *K*_D_ = 13.7 µM for KLHL20^Kelch^, based on a similar experimental setup [9]. However, the previously published data did not appear to be complex and a steady‐state analysis assuming a 1:1 interaction appears to describe it well. This difference may be due to differences in the experimental procedure, e.g., longer injection times (ca. 90 s vs. 30 s) allowing steady state to be reached, or because the binding mode is fundamentally different. Our mechanistic analyses were optimised to capture the complexities in the early stages of the interaction, and therefore typically used 15 s injections which did not allow steady state to be reached.

Nevertheless, the computational modelling of the interaction between the ZFTA peptide and KLHL20^Kelch^ assumed that it bound in a similar manner to DAPK1. It guided the rational design of a shorter helical peptide and identified residues deemed as critical for protein interaction. This allowed the prioritisation of which peptides were of interest to synthesise and helped identify residues suitable for stapling.

The experimental analysis of synthesised peptides allowed an evaluation of the relative importance of residues interacting with the protein and confirmed that the modelling identified critical residues, although the correlation between modelling and experiments was not very good. It was also noted that all designed peptides had considerably lower affinities than the original peptide used as a starting point in their design. This had a practical consequence since quantification of weak interactions using SPR biosensor analysis is difficult due to very rapid kinetics and the requirement of relatively high concentrations of analytes, often limited by their solubility. Here, we had the additional challenge of mechanistically complex interactions (discussed in the next section).

Both the experimental design and the data analysis procedures were optimised to enable quantification of interaction parameters. However, although the sensorgrams were of high quality, global regression analysis could not be used for estimation of kinetic parameters or affinities. Also, a steady‐state analysis based on report points lacked accuracy since it requires that report points are taken at steady state and the signal vs. concentration plot must show clear curvature, approaching saturation. The poor fit in Figure 1 illustrates the limitations in the data analysis. This indicates that the interaction is more complex than can be captured by a 1:1 mechanism. Extending the injection time was generally not helpful although steady state data in some cases could be obtained. The estimation of approximate *K*_D_‐values via the ‘pseudo‐steady state analysis’ provided useful information since the focus was on ranking peptides using data obtained in the same experiment and via the same analysis procedure, not on theoretically well‐defined kinetic parameters or equilibrium constants. This procedure allowed the evaluation of the effect of structural modifications on overall affinities. Competition experiments between peptides **1**, **2**, and **9** were run, but due to the relatively weak interactions, it was not possible to use high enough concentrations of peptides to block the binding site to observe competition (data not shown).

Unexpectedly, peptide **1** had a higher affinity than the peptides based on peptide **2**, designed to prioritise the formation of a stable helix by lengthening the N‐terminal region and shortening the C‐terminal region. By a comparison of our model to the crystal structure of DAPK1 in complex with KLHL20^Kelch^, it was noted that peptide **2** did not have a residue in the C‐terminal region that probed into the binding pocket as the leucine does in the critical “LPD**L**V” DAPK1 motif [9]. In addition, the effect of stapling the peptide on binding affinity was not as strong as anticipated, especially when considering the large effect that was found for a macrocyclic DAPK1 derivative, which decreased the *K*_D_ from > 5 µM to 600 nM [10]. It can therefore be hypothesised that the nonstapled peptide **2** has a helical structure whose stability is largely unaffected by stapling, and increased affinity is not achieved by further extension of the helix. Importantly, the introduced staple appears to be in a site which ultimately does not exert influence in its interaction between the peptide and KLHL20^Kelch^. The removal of the N‐terminal residues in the original peptide, resulted in a shorter peptide (**9**) with higher affinity than the initially designed N‐terminal peptide (peptide **2**). This was unexpected considering the low reliability of the model predicted by AF2 for the disordered C‐terminal peptide.

The observed complexity in the sensorgrams could not be resolved by using any of the standard interaction models in the regression analysis e.g. the basic 2‐state model (Scheme 1 in Figure S7). Alternative models considered (see Schemes 2 and 3 in Figure S7) could not be evaluated via regression analysis since they are currently not available in the software used for data analysis and the analysis of such complex models would require large datasets.

Time course experiments were therefore performed to exclude that the complexity was a result of conformational changes induced by the interaction [18, 19, 20]. Since the length of the injection did not influence the interaction kinetics, it could be concluded that time is not a factor for the complexity and that peptides dissociate in one step and a single conformation.

Although the type of complex interactions observed here are common in literature, to the best of our knowledge, no studies have established a plausible model or analytical procedure that could guide us in our interpretations. For example, the SPR biosensor data for the binding of Nrf2 to KEAP1, another Kelch domain protein, looks very similar to that shown here [11]. But no fits from the global regression analysis are shown, which is essential if the reported parameters are to be trusted.

**FIGURE 9:**
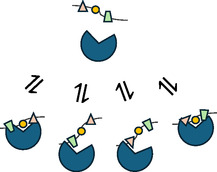
Schematic of fuzzy binding mechanism illustrated with a peptide containing multiple potential binding sites that can be aligned and oriented differently. The possibility of rearrangements without dissociation and rebinding is not shown.

Taken together, the most plausible model for peptides interacting with KLHL20^Kelch^ is ‘fuzzy binding’, as depicted in Figure 9. The model assumes that the substrate binding site can interact with the same peptide in several possible ways resulting in a heterogeneous ensemble of complexes that may potentially rearrange without dissociating first (not shown in the Figure). This infers that productive binding is not driven by specificity for a certain sequence motif, but for a structurally disordered peptide.

This model is consistent with the finding that KLHL20^Kelch^ had similar affinities for peptides representing the C‐and N‐terminal parts of a longer peptide, only overlapping by three residues (‐GGR‐). The protein thus appears to bind shorter regions of a peptide independently in multiple binding events rather than a longer peptide in one single binding event. This could explain why modelling of structures of KLHL20^Kelch^ peptide complexes was not successful for the longer peptide, while for shorter peptides it resulted in more confident conformations.

The fuzzy binding mechanism for peptide interactions with KLHL20^Kelch^ is also consistent with the lack of success with our extensive crystallisation trials. Although three crystal structures of KLHL20^Kelch^ are available in the Protein Data Bank, the published procedure for the crystallisation of the protein in complex with DAPK1 (6GY5) [9] did not work in our hands, while none of the methods for the apo‐structures (5YQ4 and 8CIA) have yet been disclosed. This suggests that establishing reliable conditions resulting in a homogeneous sample suitable for crystallography is difficult for this structurally dynamic protein. In addition, this project experienced unusual difficulties in reproducing biosensor data, even for controls. Particular care was therefore taken in storage and handling of the peptides, and detailed comparisons of peptides were only done using data from the same experiment. These difficulties are attributed to the rather flexible loops in the substrate binding site, resulting in a heterogeneous population of the target.

Importantly, the fuzzy binding model aligns with the biological function of Kelch domain of KLHL20, in which it binds disordered protein substrates for degradation, but without a well‐defined sequence specificity. The observation that ZFTA may be a substrate specific for KLHL20 is noteworthy, although its biological relevance remains to be established. Since ZFTA does not share any sequence motif with the previously known substrate DAPK1, it can be assumed that substrate recognition by KLHL20^Kelch^ is based on structural features not evident from the protein sequence, as would be expected for fuzzy interactions.

This is logical since this domain acts as an adaptor for Cul3 E3 ubiquitin protein ligase and needs to be specific for proteins with disordered regions targeted for ubiquitin mediated degradation [3, 4]. Moreover, since this interaction must be transient in order to catalyse the ubiquitination of the target protein, it cannot exhibit a very high affinity. It can therefore be assumed that the druggability of this binding site is poor, questioning the suitability of KLHL20 and perhaps other proteins with Kelch domains as druggable targets.

Although the structurally flexible binding sites and binding partners are likely to result in slow association rates and low affinities, the overall affinity may be higher since the ensemble of complexes results in an increased local concentration of peptides.

## 4 Conclusion

Taken together, this study has revealed remarkable features of the recognition and binding mechanisms for a Kelch‐like protein involved in binding disordered substrates. The SPR biosensor‐driven approach was very efficient and informative, especially considering the difficulties in modelling and experimentally determining the structure of this structurally dynamic target. However, due to the weak affinities and complex interactions of the studied peptides, innovative experimental design and data analysis procedures were required. By resolving the mechanistic basis for the complexities, data suitable for guiding the computational design process was ultimately obtained. The results highlight the challenges in predicting changes in affinity for a peptide when the target protein has flexible loops in the binding site. But we found the AF2‐based strategy in combination with rigorous SPR biosensor analysis to be considerably faster and simpler than both crystallography and NMR, neither of which resulted in useful data within the course of the project. It was ultimately realised that KLHL20 is a poorly druggable target.

More importantly, the study is expected to provide new insights into fuzzy interactions and the biological significance of weak, transient interactions with disordered proteins. Clearly there is a need for new methods and strategies for studying and describing relevant features of fuzzy interactions, beyond standard kinetic and equilibrium parameters, and static three‐dimensional structures. Here it was demonstrated that by using a variety of experimental and data analysis approaches, SPR biosensor analysis can provide useful information about fuzzy interactions.

## 5 Experimental

### 5.1 Protein Production

KLHL20^Kelch^ was expressed and pelleted by the Protein Purification Unit at Copenhagen University Centre for Protein Research using the plasmid constructs described in Supplemental information. Cell pellets were resuspended in lysis buffer (100 mM Tris, 300 mM NaCl, 1 mM MgSO_4_, 0.5 mM TCEP, 1x complete benzonase tablet pH 7.5) and sonicated. The lysate was clarified via centrifugation and filtered using a 0.7 μm filter followed by 0.45 μm filter. Lysate was applied to 2 × 5 mL GST affinity columns (GE Healthcare, Ref. 17‐5281−01) in series using binding buffer (50 mM Tris, 300 mM NaCl, 0.5 mM TCEP, 10% glycerol pH 7.5). A gradient elution was applied using binding buffer and elution buffer (same as binding buffer but with 20 mM reduced glutathione and pH 8.5) at 0.5 mL/min over 40 column volume (CV). The elution peak fractions were pooled and cleaved using 226 μM TEV (synthesised inhouse), 4°C, 22 h. TEV cleavage occurred alongside simultaneous dialysis. Dialysate was applied to a Ni^2+^‐NTA column (1 mL/min) and gradient elution run using binding buffer (50 mM Tris, 300 mM NaCl, 10 mM imidazole, 0.5 mM TCEP, 10% glycerol pH 7.5) and elution buffer (the same as binding buffer but pH 8) at 2 mL/min over 20 CV. Selected IMAC fractions were polished using gel filtration (GF). Pooled fractions were loaded onto a GF200 16/600 column and isocratically eluted over 1.2 CV at 0.5 mL/min). Eluted peak fractions were pooled and concentrated to > 2 mg/mL.

KLHL12^Kelch^ was produced from a construct encompassing residues 268–568 of KLHL12 and a Twin Strep‐MBP tag, expressed from a pCPR00183 vector in HEK293‐6E (See Supporting Information for sequences of constructs). The cells were grown in FreestyleTM F17 Expression Medium (Gibco, supplemented with 4 mM glutamine, 1% FBS, 0.1% pluronic acid, and 50 µg/mL G418) at 37°C, 140 rpm, 5% CO_2_ atmosphere in 1000 mL square polycarbonate storage bottles with 0.2 µm vented caps (Corning) and split every second day. For transfection, cells were diluted in four square bottles to a final concentration of 1 × 10^6^ cells/mL in a total of 500 ml (per 1000 mL bottle) and transfected with a total of 2 mg of plasmid DNA and 4 mg of 40 kDa polyethylenimine (PEI) diluted with Opti‐MEM I + GlutaMAX (Gibco). 2Terese Bergfors) for support during the crystallisation process4 h post‐transfection, cells were treated with 3.75 mM valproic acid to suppress cell growth and were incubated an additional 24 h and then harvested.

Twin‐Strep‐MBP‐KLHL12^268–568^ was purified by lysing the cells in 40 mL lysis buffer (350 mM NaCl, 100 mM Tris pH 8.0, 0.05% NP‐40) supplemented with complete Mini Protease Inhibitor Cocktail (Roche), PhosSTOP Phosphatase Inhibitor Cocktail (Roche), and 1 mM phenylmethylsulfonyl fluoride (PMSF). Following centrifugation, the lysate was filtered through a 0.45 μm syringe filter and was incubated with Strep‐tactin (IBA) beads for an hour at 4°C with rotation. The beads were washed three times with 1 CV lysis buffer and then an additional two times with 10 CV lysis buffer with a 10‐minute incubation with rotation at 4°C each time. After the final wash, the beads were moved to a pre‐equilibrated column and allowed to settle before eluting six times with 0.5 CV elution buffer (lysis buffer supplemented with 2.5 mM desthiobiotin pH 8.0), each time allowed to incubate 1 minute with column stopper before collecting fraction into 1.5 mL tube. Eluted fractions containing Strep‐MBP‐KLHL12^268‐568^ were identified by Instant Blue Stain and pooled.

### 5.2 Peptides

Peptides representing residues 1329–1349 of DAPK1 (LLAMNLGLPDLVAKYNTSNGA), residues 426–441 of ZFTA (YLMELDGGRRGLVCGV), and the N‐ and C‐terminal fragments thereof (RLEYLMELDGGR and GGRRGLVCGV, respectively) were purchased from GenScript. Stapled ZFTA (peptide **8**) was bought from Biosynthan (Robert‐Rössle‐Str. 10, D‐13 125, Berlin, Germany). Purchased peptides were all equipped with an N‐terminal acetyl group and a C‐terminal amide. For the native peptide, the stock was 7.7 mM and for the extended version it was 8.4 mM. Peptides were routinely solubilised in 100% DMSO to 10 mM. The concentration assumed the peptide molecular weight was a sufficient estimate and disregarded the contribution of trifluoroacetic acid (TFA) salts.

### 5.3 Peptide Synthesis

Peptides **3**‐**7** were synthesised in‐house by Fmoc‐based solid‐phase peptide synthesis using a pre‐loaded Fmoc‐RINK‐Amid‐MBHA resin (0.69 mmol/g, 100 ‐ 200 mesh, Iris Biotech), O‐(benzotriazol‐1‐yl)‐N, N,N’, N’‐tetramethyluronium hexafluoroophosphate (HBTU)/diisopropylethylamine (DIPEA) for couplings, and anhydrous DMF as solvent. All amino acids were from Novabiochem (Merck KGaA, Darmstadt, Germany) and used without further purification. Each coupling was carried out for 1 h at room temperature with resin/Fmoc‐amino acid/HBTU/DIPEA (1:5:5:10). Fmoc‐deprotection was carried out in 20% piperidine in DMF for 10 min twice, followed by DMF wash. The peptide synthesis was monitored every third amino acid by treating a sample of the resin with TFA/H_2_O for 30 min at room temperature and injecting a sampling of the supernatant in the analytical high‐performance liquid chromatography (HPLC)/electrospray ionisation (ESI)‐MS. Upon synthesis completion, all N‐terminus were acetylated, using acetic anhydride/DIPEA (10:10) in anhydrous DMF. Each acetylation coupling was carried out for 1 h at room temperature, followed by DMF, DCM, MeOH, and DCM washings, respectively. Cleavage was carried out along with protective groups deprotection (tert‐butyl and 2,2,4,6,7‐pentamethyldihydrobenzofuran‐5‐sulphonyl (PbF)) by treating the resin with TFA/triethylsilane/H_2_O (95:5:5) for 2 h at room temperature, resulting in free peptides with C‐terminal amides. This was followed by filtration, washing of the resin with TFA, concentration by nitrogen flow, precipitation with cold diethyl ether, lyophilisation, and purification with preparative reversed‐phase HPLC (RP‐HPLC).

Preparative RP‐HPLC was performed by UV‐trigged (214 nm) fraction collection with a Glison HPLC system using a Machery‐Nagel NUCLEODUR C18 HTec column (21 × 125 mm, particle size 5 µm) and H_2_O/CH_3_CN/0.1% TFA as mobile phase at a flow rate of 25 mL/min. Synthesised compounds were analysed for purity (>95%) (UV 214 nm) by analytical HPLC/ESI‐MS performed using ESI and a Penomenex Kinetex C18 column (50 × 3.0 mm, 2.6 µm particle size, 100 Å pore size) with CH_3_CN/H_2_O in 0.05% aqueous HCOOH as mobile phase at a flow rate of 1.5 mL/min. All chemicals and solvents were purchased from Sigma Aldrich, Fisher Scientific, and VWR, and used without further purification if nothing else stated.

### 5.4 Modelling of Peptide–Protein Complex

The peptide–protein complexes were modelled with AlphaFold‐multimer‐v3 and default parameters, using localcolabfold v1.5.2 [15]. Models used for visualisation and computational alanine scanning were relaxed with AMBER, using default parameters of the localcolabfold package. Computational alanine scanning was performed using a local installation of the Robetta alanine scanning implementation [16].

### 5.5 SPR Biosensor Interaction Analysis

Experiments were performed using a Biacore T200 system (Cytiva, Uppsala, Sweden). All experiments relied on amine coupling as a method for immobilisation. DMSO concentration was maintained at 1% or 2% within the following solutions across each assay: peptides and system running buffer.

KLHL20^Kelch^ was immobilised on two channels of a Biacore (Cytiva, Uppsala, Sweden) or Xantec biosensor chip (XanTec bioanalytics GmbH, Darmstadt, Germany) using amine coupling. The reference channel did not undergo protein immobilisation nor amine coupling. Protein was immobilised at 0.1 mg/mL in 50 mM sodium acetate pH 5 at 25°C using a flow rate of 5 µL/min, contact time 200 s. Protein immobilisation levels were ca. 7–11 kRU. If required, one of the two protein immobilised channels was unfolded using a single 600 s injection of 50 mM NaOH, flow rate 30 mL/min. It was observed that unfolding of the protein surface was uniform and consistent between assays.

Post immobilisation, the system temperature was lowered to 15°C. Assay buffer was either 1 x HBS, 0.05% Tween‐20 + 1% DMSO (used for assays where the top peptide concentration was 50 µM) or 1 x HBS, 0.05% Tween‐20 + 2% DMSO (used for assays where the top peptide concentration was 200 µM).

Peptides were diluted to the desired top concentration of either 50 or 200 µM using 1 x HBS, 0.05% Tween‐20. Twofold dilutions were made for each peptide (for a total of 7 (50–0.8 µM) or 9 (200–0.8 µM) concentrations respectively) using the respective assay buffer and tested in dilution series. Peptide injection time was either 15 s or 60 s, whilst dissociation remained at 15 s for both assay types. After each peptide cycle the injection needle was rinsed in 50% (v/v) DMSO.

Analysis was conducted using the Biacore Evaluation software. Solvent correction and reference subtraction were conducted.

### 5.6 Analysis of Alanine Scanning ZFTA Derived Peptide Variants

Immobilisation and denaturation of KLHL20^Kelch^ was carried out as previously stated, however, the concentration of DMSO within the running buffer was kept at 2% to allow for an increased concentration of peptide variants to be tested. Post immobilisation temperature was lowered to 15°C. Assay buffer was 1 × HBS, 0.05% Tween‐20 + 2% DMSO. Flow rate was 30 µL/min. Peptides were tested following a concentration series. Alanine scanning variants **4**–**7** along with peptide **2** had a maximal concentration of 200 µM. All other peptides had a maximal concentration of 50 µM. Peptides were diluted in 1 × HBS + 0.05% Tween with subsequent 2‐fold dilutions made using 1 x HBS + 0.05% Tween + 2% DMSO. Two zero concentrations of 1 x HBS + 0.05% Tween + 2% DMSO were included at the beginning and the end of each compound series.

### 5.7 Investigation of Effect of Contact Time on Dissociation Rate

Immobilisation of KLHL20^Kelch^ was carried out as before at 25°C, this time no surface was denatured. Post immobilisation temperature was decreased to 15°C. Assay buffer was 1 x HBS, 0.05% Tween‐20 + 2% DMSO. Flow rate was 30 µL/min. Peptides were tested at a single concentration of 200 µM, aside from peptide **8**, which was 50 µM. Contact time of the peptide injection was: 15, 30, 45, 60, 75, 90, 120, and 140 s. The dissociation period remained constant at 15 s.

### 5.8 Thermal Shift Analysis

KLHL20^Kelch^ was diluted to 0.2 mg/mL in 50 mM sodium acetate pH 5. DAPK1 peptide (10 mM) and ZFTA peptide (**1**) (10 mM) were diluted to 200 and 100 µM, respectively in 1 × HBS, 0.05% Tween‐20. An equal volume of protein and peptide were mixed together and from this mixture a capillary tube was used to take the sample (ca. 10 µL) for thermal shift measurement. Thermal shift analysis was conducted between 35 and 95°C using a Tycho instrument (NanoTemper, Münich, Germany).

### 5.9 Protein Crystallography

Two simultaneous routes were followed, which included replicating the reported conditions from literature [1, 9] as well as using the most promising conditions from the thermal shift analysis. Two trials were based on the latter approach: Protein was concentrated to ca. 10 or 34 mg/mL, using a 10 kDa MWCO concentrator. The concentrated solution was centrifuged to pellet any precipitate and aliquoted into separate tubes before incubating with ligands for 10 min. The ligand concentration was in excess to protein concentration to ensure binding site saturation (protein : ligand ratio = 1:1.5). *Apo* and *holo* protein crystallisation conditions were prepared on MRC3 96‐well plates using molecular dimensions crystallisation screening kits of MemGold I, MemGold II, PACT Premier, and JCSG Plus. The formation of transparent crystals was typically observed under elevated salt concentration conditions such as 1) 0.2 M sodium acetate trihydrate, 0.2 M potassium chloride, 0.1 M HEPES, pH 7.0; 2) 0.3 M magnesium nitrate hexahydrate, 0.1 M Tris, pH 8.0; or 3) 0.2 M ammonium chloride, 0.1 M sodium acetate, pH 5.0, 20% PEG 6000, which is similar to conditions reported in the literature. Crystal hits from these conditions and those from literature were further optimised and grown in a crystallisation hotel (Rock Imager) at 20°C over 4 weeks. Crystals large enough and of sufficient quality were harvested in the mother liquor and sent for data collection at the diamond synchrotron light source (Didcot, UK). Despite coating each sample in different cryoprotectants such as glycerol, polyethylene glycol (PEG), and 2‐methyl‐2,4‐pentanediol (MPD), only very low‐resolution diffraction was observed (~8 Å).

### 5.10 Protein Observed NMR

Double isotopically labelled (^15^N and ^2^H) KLHL20^Kelch^ was produced for NMR at the Lund Protein Production Platform (Lund University, Lund, Sweden) in *E. coli* using a protocol based on a previously described protocol for labelling [21]. It used a pNIC28‐Bsa4 plasmid and *E. coli* TUNER (DE3) for expression. The purification involved IMAC, TEV cleavage for removal of His‐tag, reverse IMAC, and gel filtration. Details of the expression construct and protein production can be found in the Supporting Information.

The NMR experiment was conducted with 79 µM of isotopically labelled KLHL20^Kelch^ in 50 mM HEPES (pH 7.5), 300 mM NaCl, 0.5 mM TCEP, and 10% D_2_O (190 µL). The peptide was titrated into the sample at a ratio of 1:0, 1:1.3, 1:3.3, and 1:6.6. Measurements were conducted on a 900 MHz Bruker NMR magnet equipped with a 3 mm TCI probe (Swedish NMR Centre in Gothenburg, Sweden).

## Author Contributions

**Nadine E. M. Myers**: study design, SPR biosensor experiments, thermal shift experiments, production of KLHL12^Kelch^ and KLHL20^Kelch^, X‐ray crystallography, writing – original draft, writing – review and editing. **Joanna Whittaker**: X‐ray crystallography, peptide purity controls, thermal stability analysis, and SPR biosensor experiments. **Marie** **Elodie Hélène Cadot**: synthesis of peptides, writing – original draft. **Julia K.** **Varga**: computational modelling and peptide design, writing – original draft. **Marcel** **Diallo**: production of KLHL12^Kelch^ and KLHL20^Kelch^. **Jakob** **Nilsson**: supervision of KLHL20^Kelch^ production. **Anders Bach**: supervision of peptide synthesis. **Anja Sandström**: supervision of peptide synthesis**.** **Ora Schueler‐Furman**: supervision of computational modelling and peptide design, writing ‐ review and editing**. U. Helena Danielson**: conceptualisation, funding acquisition, supervision, writing – review and editing.

## Supporting Information

Additional supporting information can be found online in the Supporting Information section. Supporting Methods: Plasmid constructs for KLHL20^Kelch^, Production of double isotopically labelled (^15^N and ^2^H) KLHL20^Kelch^ **Supporting Fig. S1**: SPR biosensor data for peptide **1** and DAPK1 peptide. **Supporting Fig. S2**: Model of peptide **9. Supporting Fig. S3**: SPR biosensor data for peptide **1** and peptide **2.** **Supporting Fig. S4**: Purity of synthesised peptides. **Supporting** **Fig.** **S5**: SPR biosensor data for N‐terminal ZFTA peptide analogues – 30 s injections. **Supporting Fig. S6**: SPR biosensor data for N‐terminal ZFTA peptide analogues – 60 s injections. **Supporting** **Fig.** **S7**: Interaction mechanisms for complex interactions. **Supporting** **Fig.** **S8**: SPR biosensor data for peptide **S8**. **Supporting** **Fig.** **S9**: SPR biosensor data for ZFTA peptide analogues – different injection times. **Supporting** **Fig.** **S10**: Thermal stability analysis of KLHL20^Kelch^ for optimisation of crystallisation. **Supporting Fig. S11**: 2D NMR of peptide **8** against isotopically labelled KLHL20^Kelch^.

## Funding

This work was partly supported by grants from European Union's Horizon 2020 Research and Innovation program under the Marie Skłodowska‐Curie Grant Agreement UBIMOTIF No 860517 (to U.H.D., J.N, O.S.‐F. and A.B.). Work at the Novo Nordisk Foundation Center for Protein Research is supported by grant NNF14CC0001. J.W. was financially supported by the Human Frontier Science Program (LT0005/2022‐C). Work at the Center for Epigenetic Cell Memory, Danish Cancer Institute is supported by The Danish National Research Foundation (DNRF195). The views and opinions expressed are only those of the authors and do not necessarily reflect those of the European Union or the European Commission. Neither the European Union nor the European Commission can be held responsible for them. Research in the O.S.‐F. lab was partially supported by the Israel Science Foundation (ISF) founded by the Israel Academy of Sciences and Humanities (grant nos. 301/2021 and 3091/23).

## Conflicts of Interest

The authors declare no conflicts of interest.

## Supporting information

Supplementary Material

## Data Availability

The data that support the findings of this study are available from the corresponding author upon reasonable request.
