# Supplementary material for: Identification of ZFTA as a Novel KLHL20 Substrate and Mechanistic Insights Into Fuzzy Binding of Disordered Peptides via Biosensor Analysis and Computational Modelling
Source: Chembiochem. 2026 Feb 28;27(5):e70237. doi: 10.1002/cbic.70237 (PMC12949624; doi:10.1002/cbic.70237)

Supplemental information

Identification of ZFTA as a novel KLHL20 Substrate and Mechanistic Insights into Fuzzy Binding of Disordered Peptides *via* Biosensor analysis and Computational Modelling

Nadine E. M. Myers, Joanna Whittaker, Marie Elodie Hélène Cadot, Julia K. Varga, Marcel Diallo, Jakob Nilsson, Anders Bach, Anja Sandström, Ora Schueler-Furman and U. Helena Danielson

Table of Contents

Methods **2**

Plasmid constructs for production of KLHL20^{Kelch} **2**

Production of double isotopically labelled (^{15}N and ^{2}H) KLHL20^{Kelch} **3**

Figures **4**

Figure S1. SPR biosensor data for peptide 1 and DAPK1 peptide **4**

Figure S2. Model of peptide 9 .... **5**

Figure S3. SPR biosensor data for peptide 1 and peptide 2 .... **6**

Figure S4. Purity of synthesised peptides **7**

Figure S5. SPR biosensor data for N-terminal ZFTA peptide analogues – 30 s injections **10**

Figure S6. SPR biosensor data for N-terminal ZFTA peptide analogues – 60 s injections **11**

Figure S7. Interaction mechanisms for complex interactions **12**

Figure S8. SPR biosensor data for peptide 8 .... **13**

Figure S9. SPR biosensor data for ZFTA peptide analogues – different injection times **14**

Figure S10. Thermal stability analysis of KLHL20^{Kelch} for optimisation of crystallisation **15**

Figure S11. 2D NMR of peptide 8 against isotopically labelled KLHL20^{Kelch} **16**

Supplemental information

Methods

Plasmid constructs for production of KLHL20^{Kelch}

The Kelch domain of KLHL20 (amino acid residues 300-609 in Uniprot entry Q9Y2M) was produced in *E. coli* from a construct with an N-terminal His_6-tag (yellow highlight), GST and a TEV cleavage site (underlined), inserted into a plasmid with kanamycin resistance (pCPR0063, $Kana^R$).

Expressed protein sequence:
MHHHHHHSSMSPILGYWKIKGLVQPTRLLLEYLEEKYEEHLYERDEGDKWRNKKFELGLEFPNLPYYIDGDVKLTQSMA
IIRYIADKHNMLGGCPKERAEISMLEGAVLDIRYGVSRIAYSKDFETLKVDFLSKLPEMLKMFEDRLCHKTYLNGDHVTHP
DFMLYDALDVVLYMDPMCLDAFPKLVCFKKRIEAIPQIDKYLKSSKYIAWPLQGWQATFGGADHPPKSSSGVDLGTEN
LYFQSMRPLMQGPRTRPRKPIRCGEVLFAVGGWCSGDAISSVERYDPQTNEWRMVASMSKRRCGVGVSVLDDLLYA
VGGHDGSSYLNSVERYDPKTNQWSSDVAPTSTCRTSVGVAVLGGFLYAVGGQDGVSCLNIVERYDPKENKWTRVAS
MSTRRLGVAVAVLGGFLYAVGGSDGTSPLNTVERYNPQENRWHTIAPMGTRRKHLGCAVYQDMIYAVGGRDDTTEL
SSAERYNPRTNQWSPVVAMTSRRSGVGLAVVNGQLMAVGGFDGTTYLKTIEVFDPDANTWRLYGGMNYRRLGGGV
GVIKMTHCESHIW

The protein was purified as a monomer. The identity of the protein was confirmed by ESI-TOF mass spectrometry (the Protein Production and characterisation platform at Novo Nordisk Foundation Center for Protein Research)

Production of double isotopically labelled (^{15}N and ^{2}H) KLHL20^{Kelch}

cDNA sequence of the expressed protein from start to stop-codon in pNIC28-Bsa4:

```
ATGCACCATCATCATCATCATTCTTCTGGTGTAGATCTGGGTACCGAGAACCTGTACTTCCAATCCATGC
AAGGACCAAGGACGAGACCACGGAAACCTATCCGATGTGGGGAAGTACTCTTTGCAGTTGGTGGTTGGTG
CAGTGGAGATGCCATTTCCAGTGTTGAACGATATGATCCACAGACCAATGAATGGAGAATGGTGGCTTCA
ATGAGCAAAAGGAGATGCGGAGTTGGGGTCAGTGTTCTTGATGATCTGTTATATGCAGTAGGAGGCCATG
ATGGATCCTCTTATCTCAATAGTGTTGAAAGGTATGACCCCAAAACAAACCAGTGGAGCAGTGATGTGGC
CCCTACAAGCACCTGCAGGACAAGTGTTGGTGTAGCAGTACTTGGAGGCTTTCTTTATGCTGTGGGTGGC
CAGGATGGTGTGTCTTGCCTCAACATTGTTGAGAGGTATGATCCGAAGGAGAACAAGTGGACTCGGGTAG
CTTCTATGAGTACCAGAAGACTAGGTGTGGCTGTGGCTGTGTTAGGAGGGTTCTTATATGCTGTAGGTGG
CTCTGACGGGACATCTCCTCTCAACACAGTGGAACGTTACAATCCTCAGGAAAACAGATGGCACACTATA
GCCCCTATGGGGACCCGGAGGAAACACCTAGGCTGTGCAGTATATCAGGACATGATCTATGCTGTAGGAG
GTAGAGATGACACTACAGAGCTGAGCAGTGCTGAGAGATACAACCCCAGAACCAACCAGTGGTCTCCAGT
GGTGGCCATGACATCACGCCGTAGTGGAGTTGGCCTGGCAGTGGTCAATGGACAGCTCATGGCAGTAGGA
GGTTTTGATGGCACAACATACTTGAAGACCATAGAAGTTTTTGATCCTGATGCCAATACATGGAGGTTAT
ATGGCGGGATGAATTACCGTCGGCTAGGGGGTGGCGTAGGAGTTATTAAAATGACACATTGTGAATGA
```

The expression plasmid was transformed into Tuner (DE3) cells (Novagen). Expression was performed in 2 x 1 L ModC1 minimal medium (Koruza et al, Arch. of biochem. and Biophys. 2018; 645:26-33) with D_2O, ^{1}H-glycerol and $^{15}NH_4Cl$. The cells were first grown in LB at 37 °C to OD_{600} =1.1. The cultivation was done in two Tunair flasks with indentations (1 L/flask) at 37 °C, 160 rpm. Then the medium was changed to ModC1 as described in (Koruza et al, Arch. of biochem. and Biophys. 2018; 645:26-33). After 1 h adaption to the new medium the cells were moved to 25 °C and another 1 h later, expression was induced by 0.2 mM IPTG at 25 °C and allowed to continue for 24 h before harvesting. The pellet was stored at -80 °C until purification. All steps were done at 4 °C if not mentioned otherwise. The cell pellet was re-suspended in wash buffer (50 mM sodium phosphate buffer, 300 mM NaCl, 20 mM imidazole, pH 8.0) to a total volume of ~30 mL. The resuspension was supplemented with a pinch of DNase I and one tablet of Complete Protease Inhibitor, EDTA-free (Roche) and passed twice through a French Pressure Cell at 18000 psi. The resulting lysate was ultra-centrifuged in a Ti 50.2 rotor, 45.000 rpm, 60 min, 4 °C. The supernatant was passed through a 0.45 µm filter and used for affinity chromatography.

Affinity chromatography with IMAC was done on 1 mL HisTrap HP column (Cytiva) equilibrated in wash buffer and elution was done with a linear gradient to 500 mM imidazole over 20 column volumes (CV). The His-tag was removed with His-tagged tobacco etch virus (TEV) protease (Lund Protein Production Platform) at 16 °C overnight. Cleaved tag and His-tagged TEV were removed from the cleaved protein by reverse IMAC in wash buffer. The flow through and wash fractions of the reverse IMAC were collected, concentrated and further purified using size exclusion chromatography (SEC) on a HiLoad 16/600 75pg SEC column (Cytiva) in 50 mM HEPES, 300 mM NaCl, 0.5 mM TCEP, pH 7.5**.** The peak fractions were pooled, concentrated to 6.8 mg/mL and buffer exchanged to SEC buffer with 10% (v/v) D_2O, and then aliquoted and snap-frozen in liquid nitrogen and stored at -80 °C.

Supplemental information

Figures

*Figure S1. SPR biosensor data for peptide **1** and DAPK1 peptide*

SPR biosensor data for the originally identified 16-mer ZFTA (peptide **1**) and a 21-mer DAPK1 peptide interacting with KLHL20^{Kelch} immobilised to the sensor surfaces. The experimental data is the same as those in the top row in Fig. 1A, but here the predicted sensorgrams from a global non-linear regression analysis of the sensorgrams using 1:1 and 2-state models are overlaid the experimental curves. The graphs show that there is no significant improvement when using a 2-state model instead of a 1:1 model. Note the different y-axes.

A) Peptide **1** and 1:1 model, K_D = 8.0 µM, B) DAPK1 and 1:1 model, K_D = 12 µM, C) Peptide **1** and 2-state model, K_D = 3.7 µM, and D) DAPK1 and 2-state model, K_D = 15 µM.

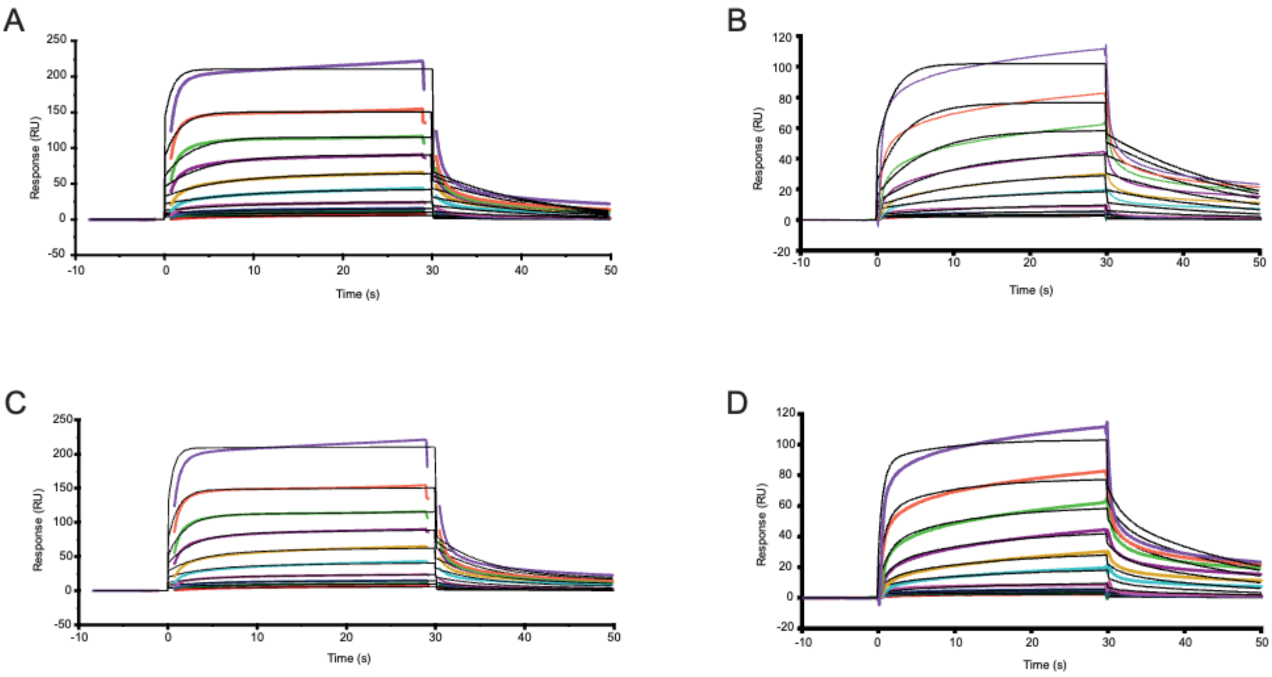

Supplemental information

*Figure S2. Model of peptide **9***

AlphaFold2 docking of the C-terminal peptide (**9**, *GGR*RG**LV**CGV), colored according to pLDDT (yellow: 50-70, orange < 50). The most confident residues are two hydrophobic residues in the peptide (LV, in bold). These side chains are shown as sticks.

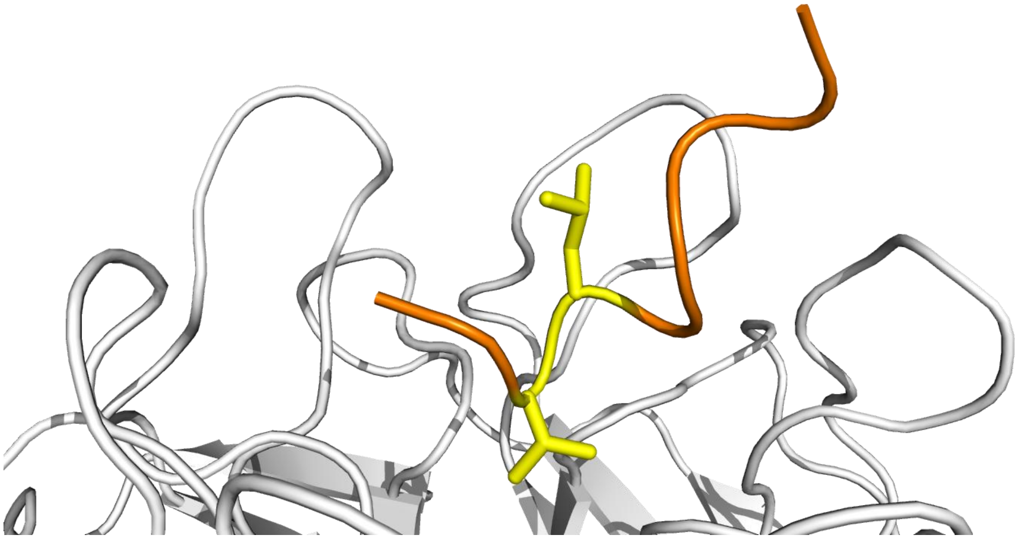

*Figure S3. SPR biosensor data for peptide **1** and peptide **2***

SPR biosensor data for original 16-mer ZFTA (peptide **1**) and peptide **2** interacting with KLHL20^{Kelch} immobilised to the sensor surfaces. The experimental data is the same as in Figure 4 (top panels). Here the lack of improvement when using a 2-state model instead of the 1:1 model for the global non-linear regression analysis of the sensorgrams is highlighted. Note the different y-axes.

A) Peptide **1** and 1:1 model, K_D =24 µM, B) Peptide **2** and 1:1 model K_D = 10 mM, C) Peptide **1** and 2-state model, K_D = 24 µM, and D) Peptide **2** and 2-state model, K_D = 2.03 mM

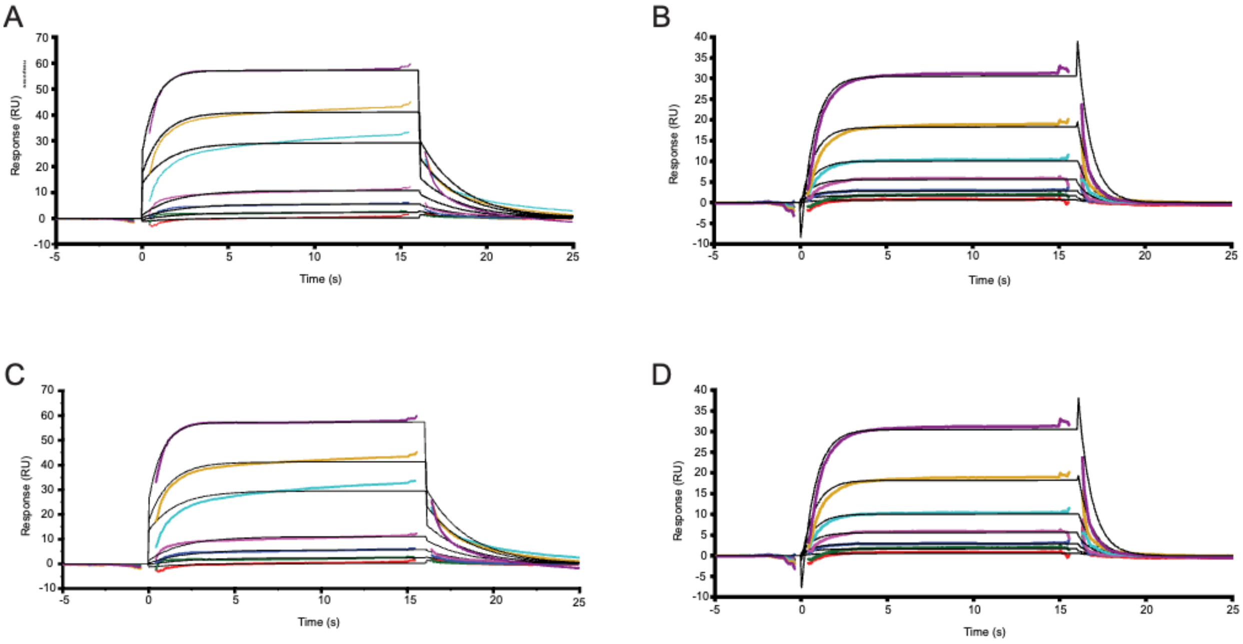

Supplemental information

Figure S4. Purity of synthesised peptides

Peptide 3

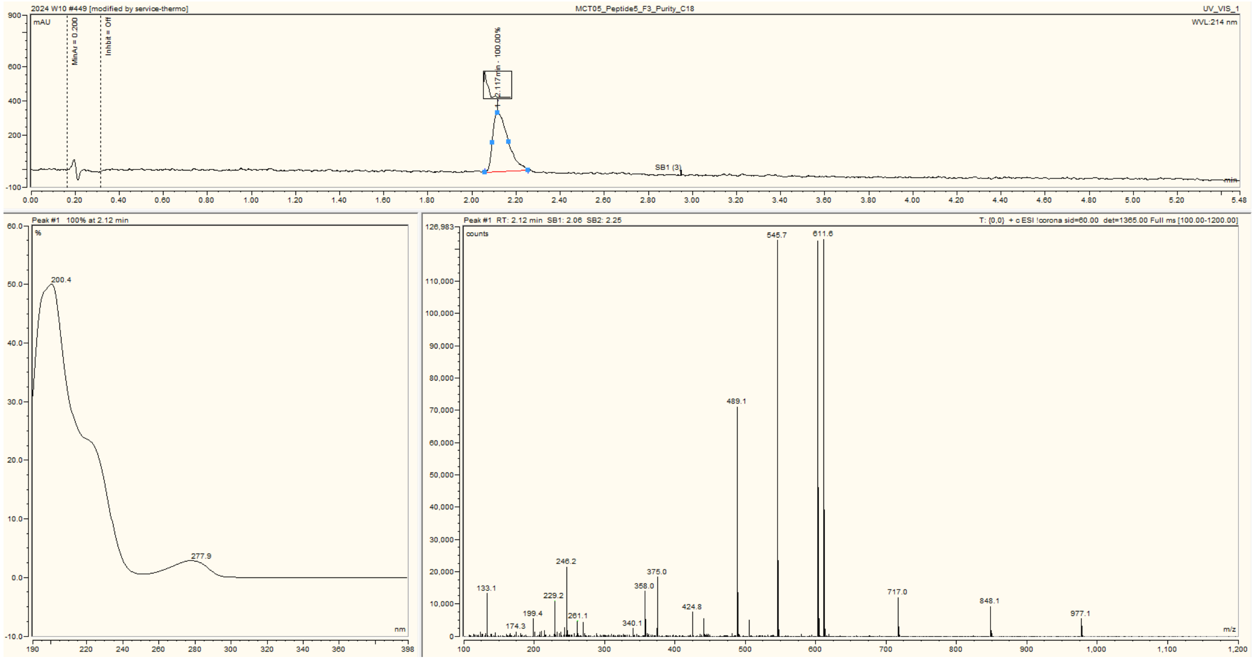

Peptide 4

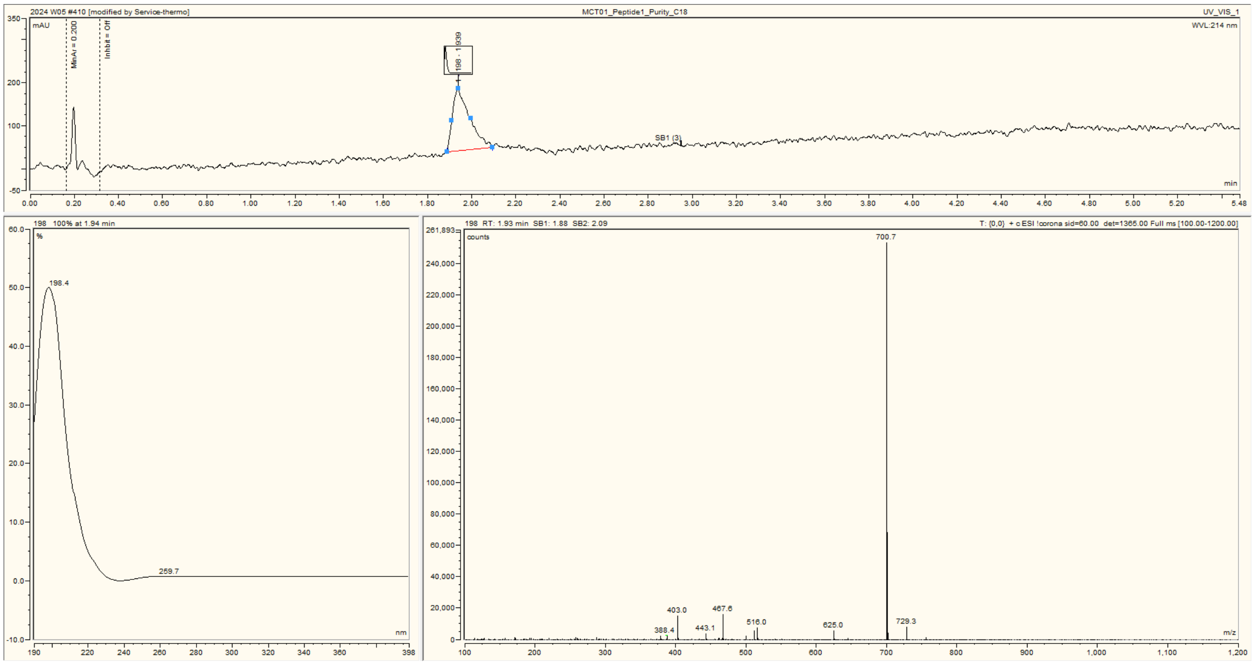

Supplemental information

Peptide 5

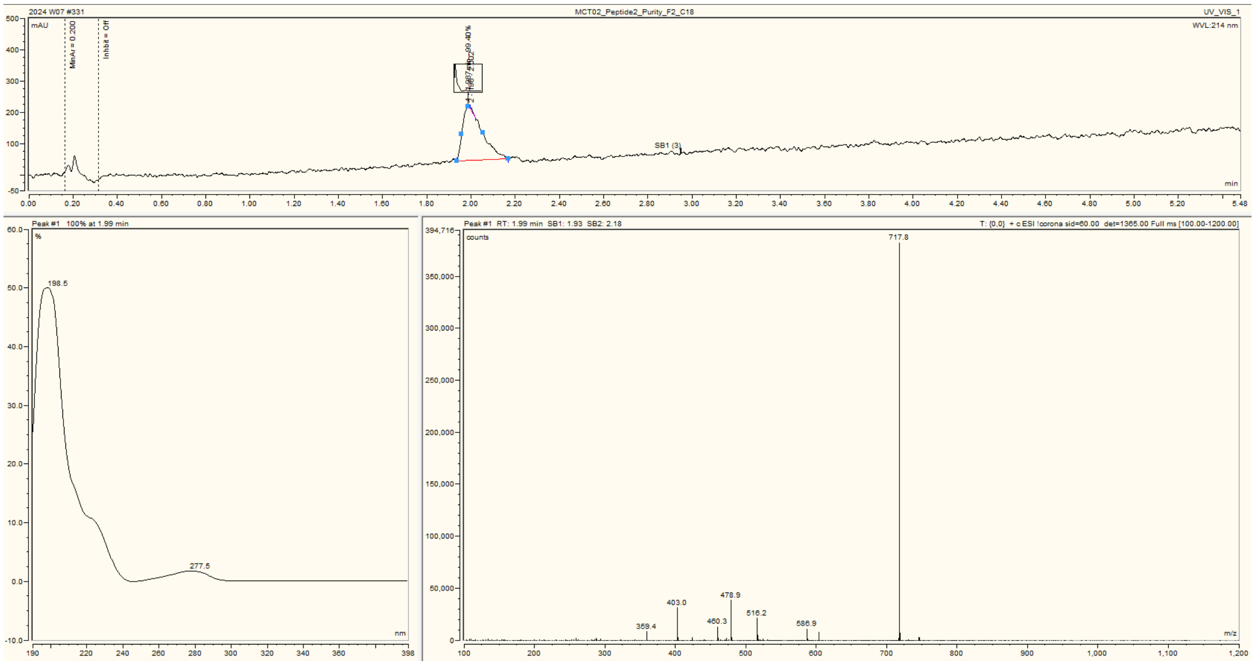

Peptide 6

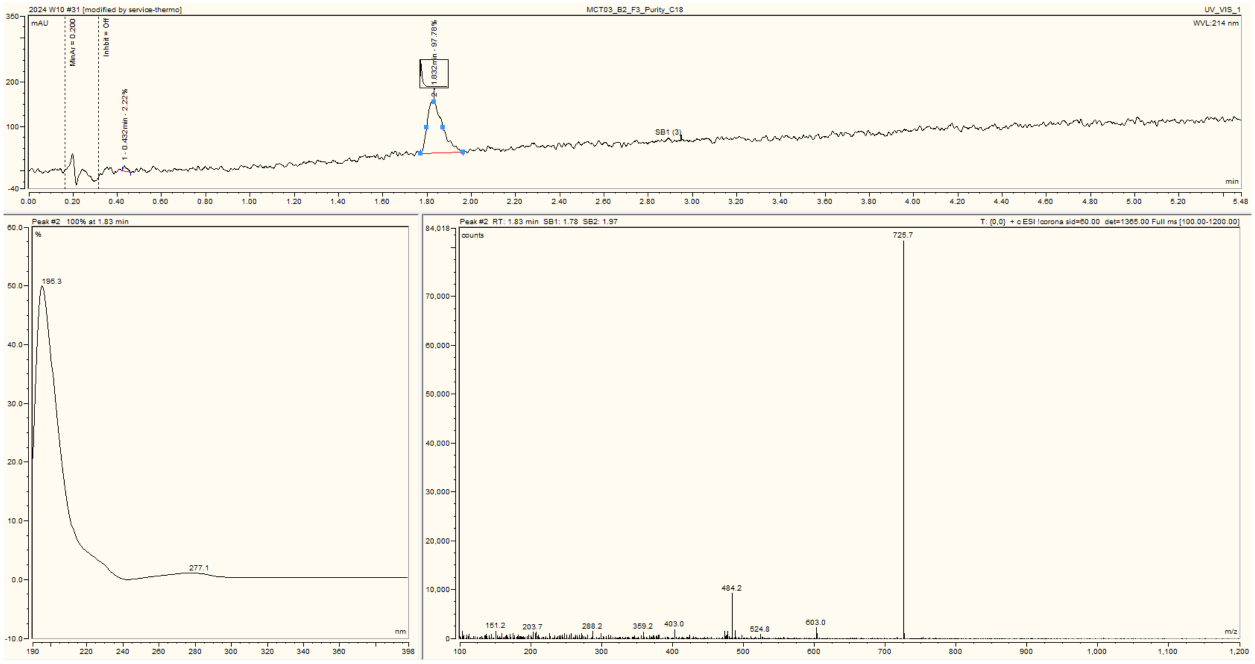

Supplemental information

Peptide 7

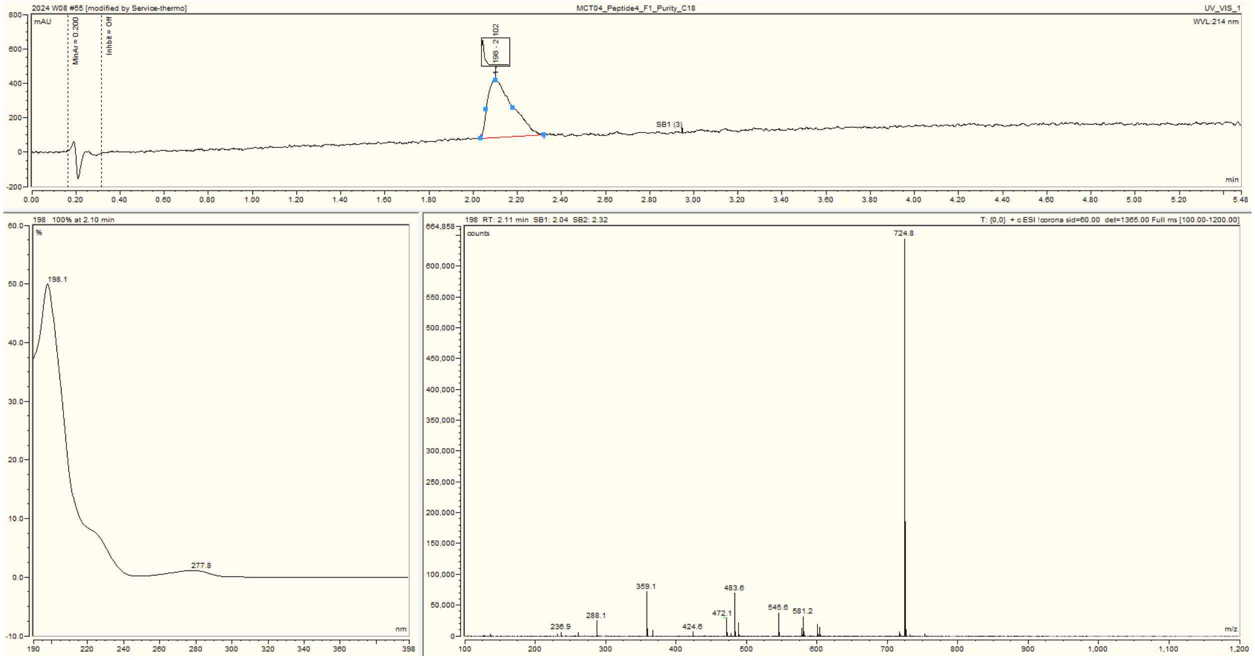

Peptide 8

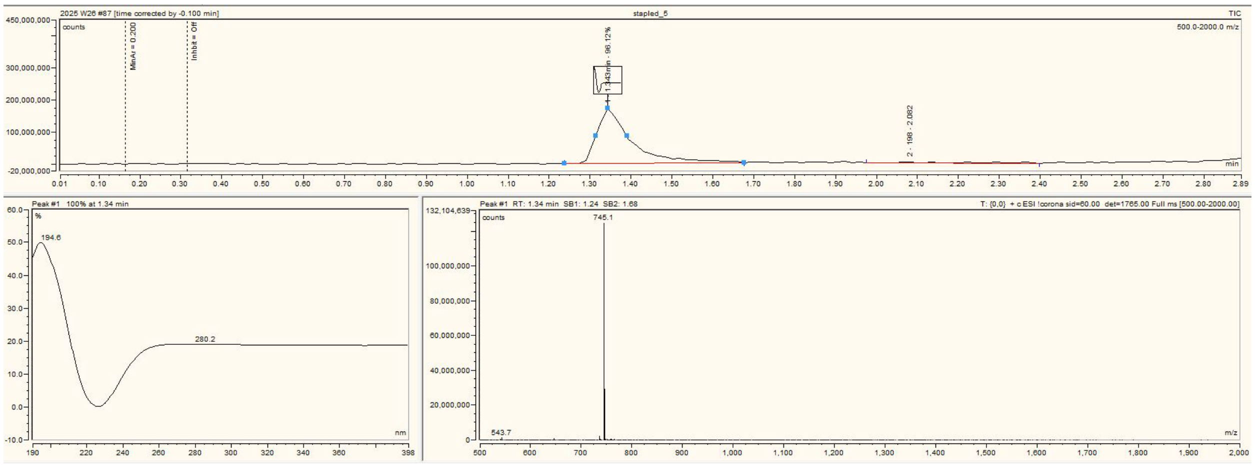

Figure S5. SPR biosensor data for N-terminal ZFTA peptide analogues – 30 s injections

SPR biosensor data for analogues of peptide **2** interacting with KLHL20[Kelch] immobilised to the sensor surfaces (as in Fig. 5). The black lines show the predicted curves from a global non-linear regression analysis using a 2-state model. Peptide **8** is stapled.

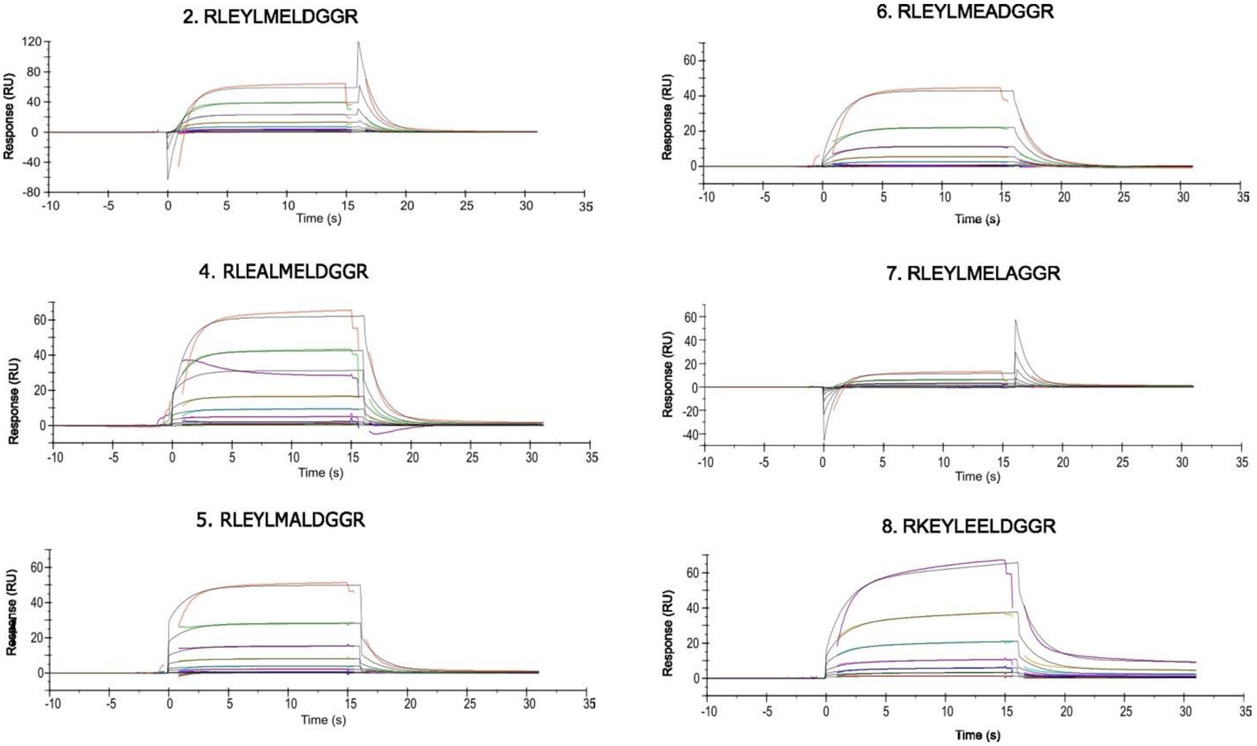

Figure S6. SPR biosensor data for N-terminal ZFTA peptide analogues – 60 s injections

SPR biosensor data for analogues of peptides **2**, and **4**-**8** interacting with KLHL20^{Kelch} immobilised to the sensor surfaces. This experiment is similar to that in Figure 5, but has here an injection time of 60 s instead of 15 s.

Blue residues represent the sequence maintained from the native ZFTA peptide, whereas the black residues represent the extended N-terminus. The alanine substituted peptides (**4**-**7**) have introduced alanine residues in red. Left) Sensorgrams for peptides injected as analytes in concentration series using 1:1-dilutions from a top concentration of 200 µM for all peptides except peptide **8** (stapled) which had a top concentration of 50 µM. Insets are graphs with signals for report points taken at the end of injections, plotted against peptide concentrations.

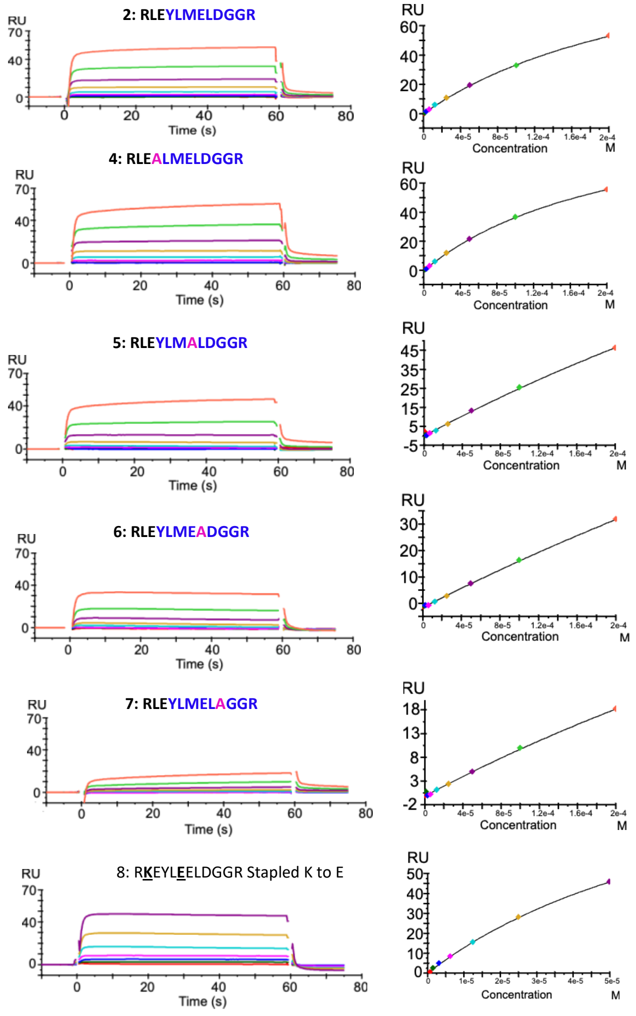

Figure S7. Interaction mechanisms for complex interactions

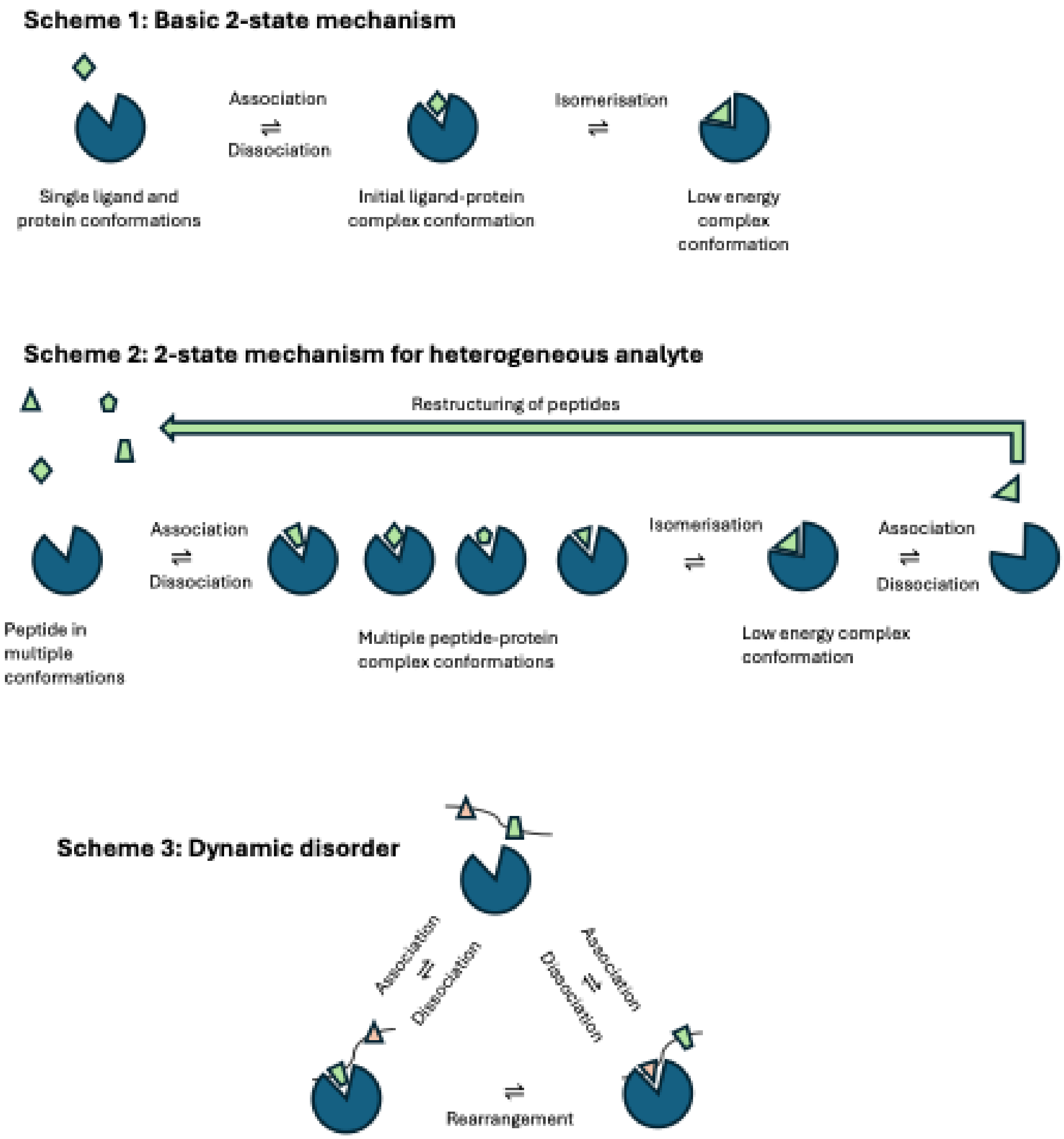

*Figure S8. SPR biosensor data for peptide **8***

SPR biosensor data for peptide **8** interacting with KLHL20^{Kelch} immobilised to the sensor surfaces injected from a 1:1 dilution series starting from a top concentration of 50 µM for 15 and 60 s (top and bottom, respectively). The data is the same as in Fig. 5 and Fig. S7, respectively. Here the lines show the predicted curves from a global non-linear regression analysis using a 2-state model.

The 2-state analysis of the data from the 15 s injection gave an approx. K_D = 1.1 mM and the 60 s injection gave an approx. K_D = 0.52 µM.

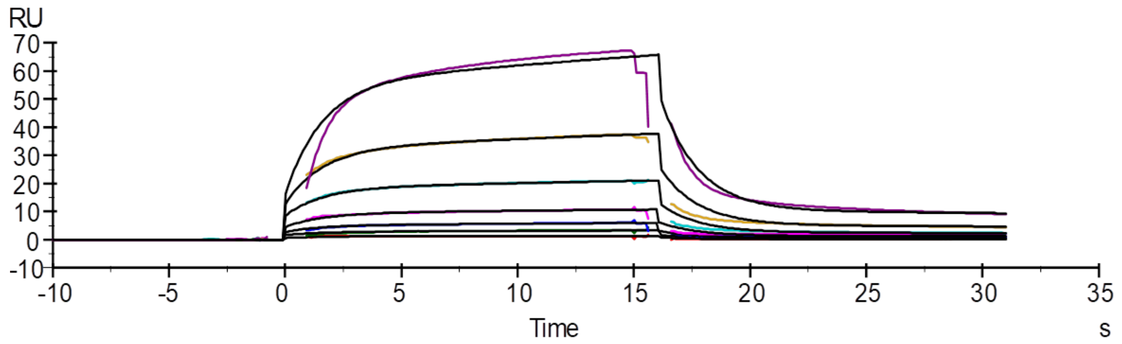

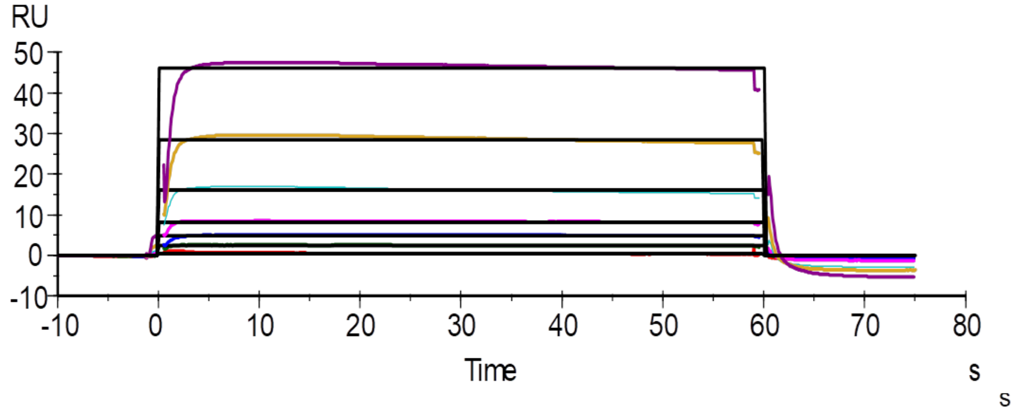

Figure S9. SPR biosensor data for ZFTA peptide analogues – different injection times

SPR biosensor data for analogues of the ZFTA peptide with an extended N-terminus (peptide **2**) interacting with KLHL20^{Kelch} immobilised to the sensor surfaces. Blue residues represent the sequence maintained from the native ZFTA peptide, whereas the black residues represent the extended N-terminus. The alanine substituted peptides (**4**-**7**) have introduced alanine residues in red. Sensorgrams for peptides injected as analyte at the same concentration (50 µM for peptide 8, and 200 µM for all other peptides) and increasing injection times (15 to 140 s), aligned from injection start. The noise after injections are instrument related artifacts and can be ignored.

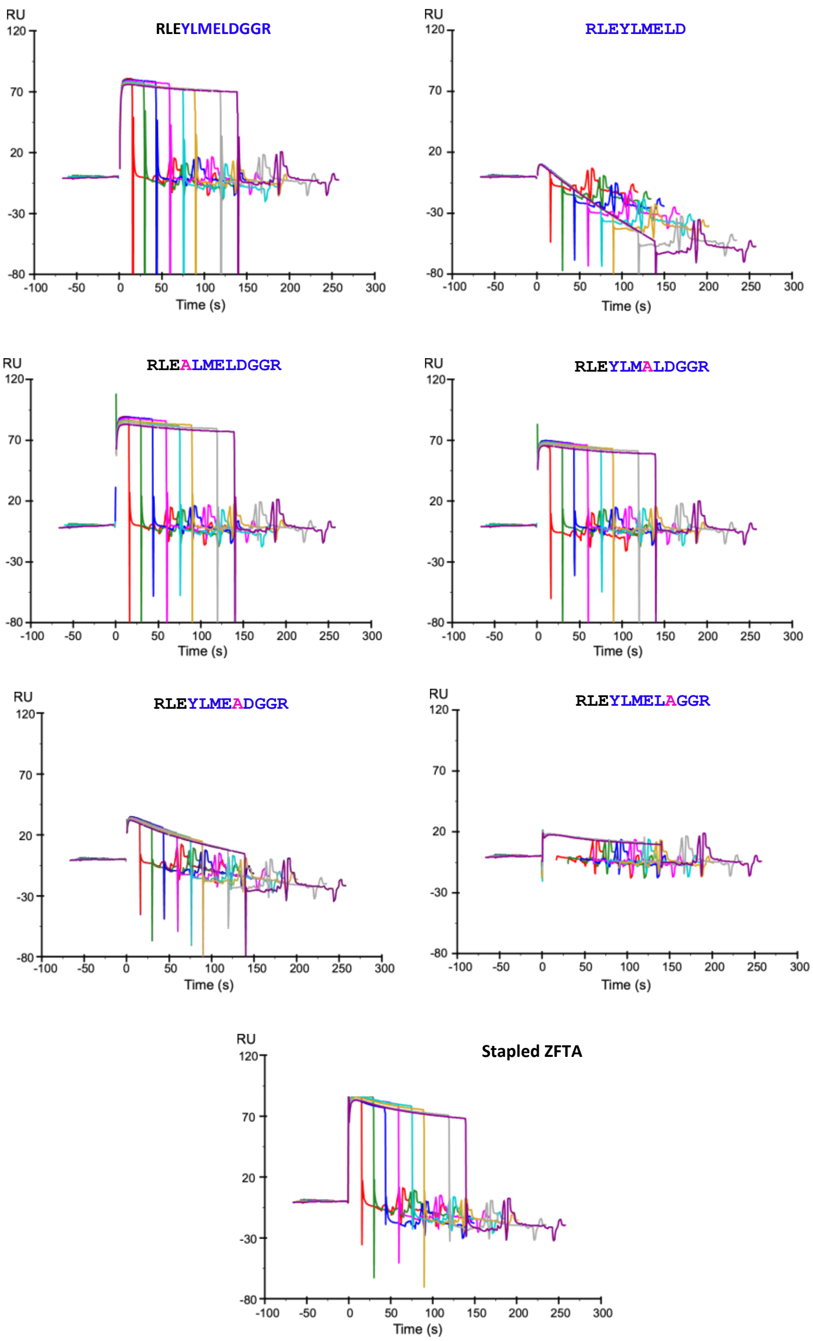

Supplemental information

Figure S10. Thermal stability analysis of KLHL20^{Kelch} for optimisation of crystallisation

Thermal stability analysis of KLHL20^{Kelch} in A) different buffers potentially suited for crystallography, B) HEPES pH 7.0 (based on a buffer screen), in the presence of different additives, and C) comparison of original and optimised buffers.

A)

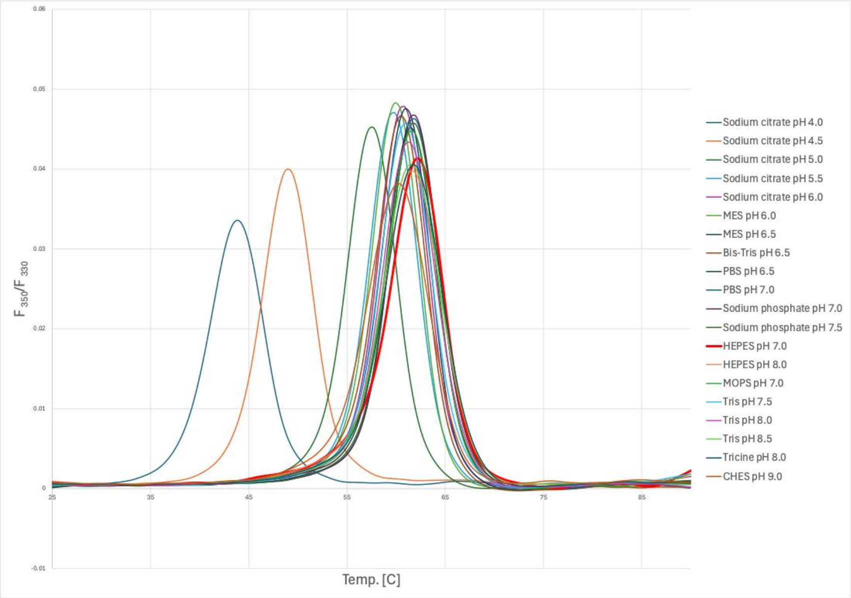

B)

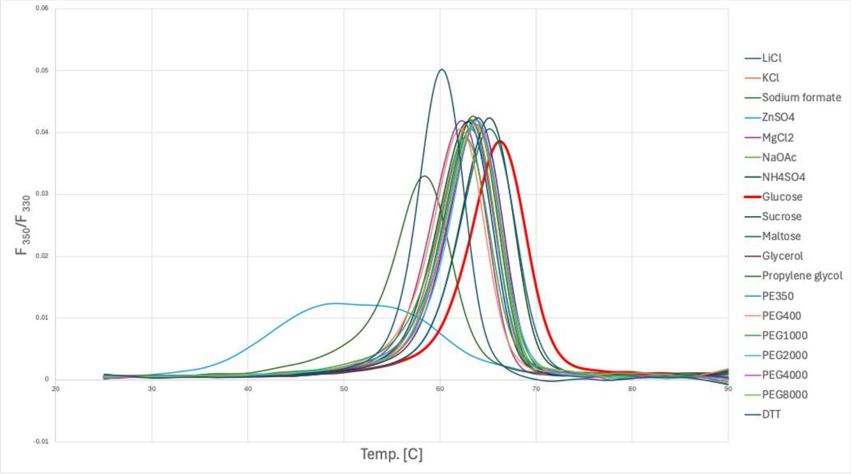

C)

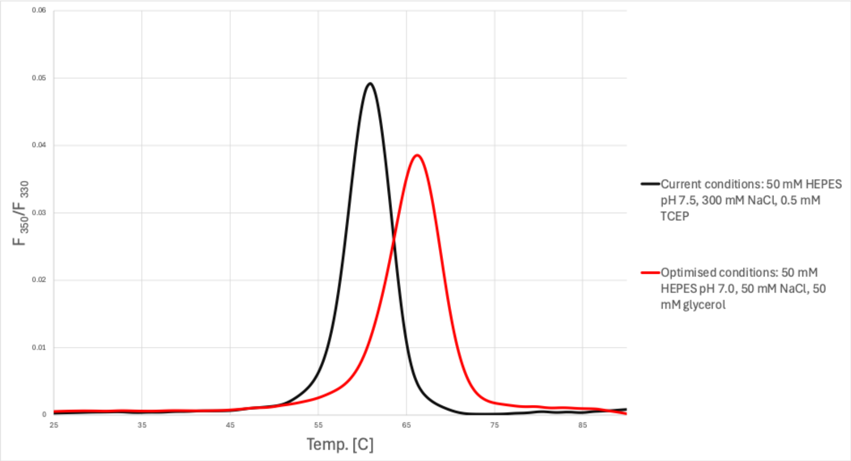

Supplemental information

*Figure S11. 2D NMR of peptide **8** against isotopically labelled KLHL20^Kelch^*

Overlay of 2D NMR spectra showing the titration of the stapled peptide (**8**) against isotopically labelled KLHL20^Kelch^ (79 µM) at a ratio of 1:0, 1:1.3, 1:3.3 and 1:6.6. Inset shows zoom-in of one of nine peaks with characteristic shift of signal positions upon titration.

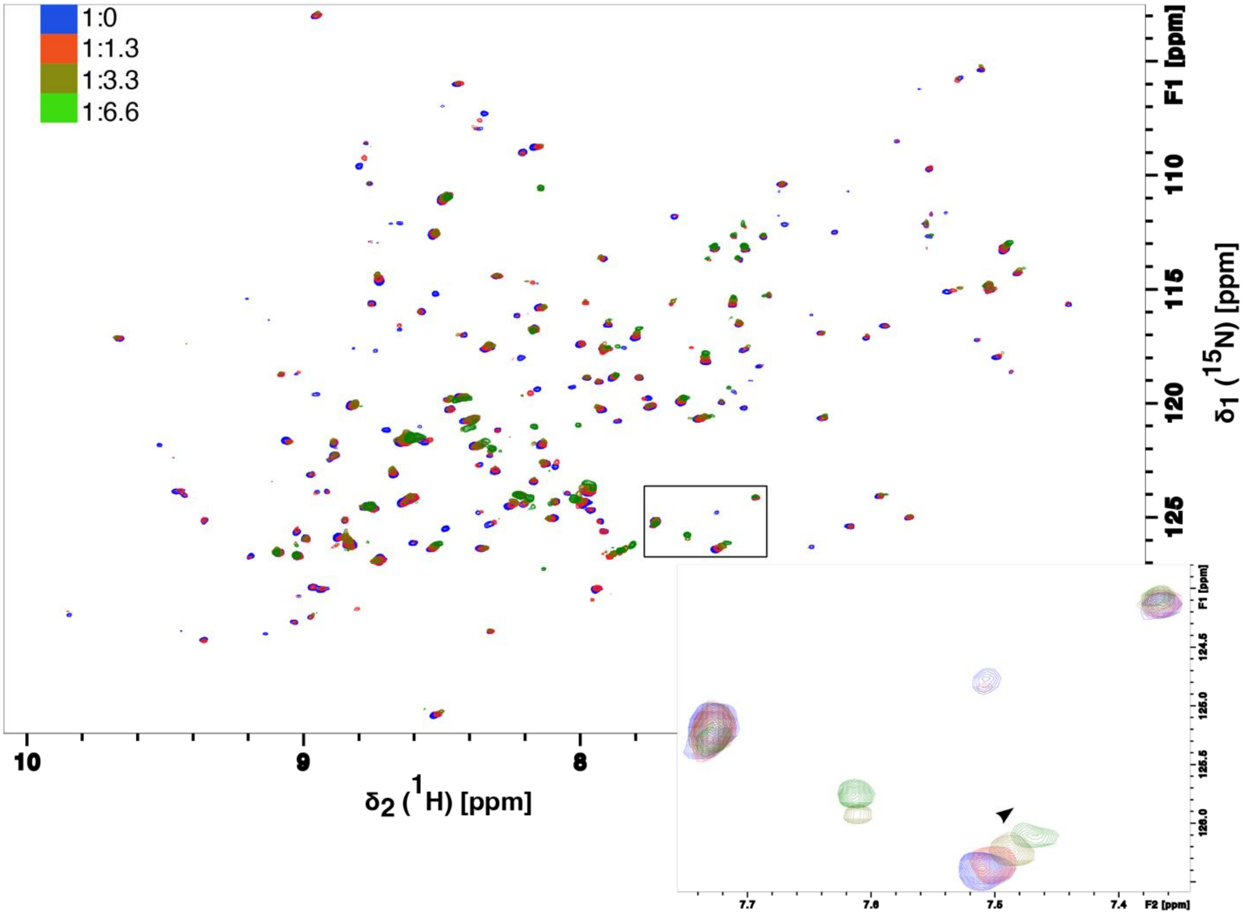

Supplement: Supplementary file 1 — Supplementary Material [file CBIC-27-e70237-s001.pdf]
